# Private management of African protected areas improves wildlife and tourism outcomes but with security concerns in conflict regions

**DOI:** 10.1073/pnas.2401814121

**Published:** 2024-07-01

**Authors:** Sean Denny, Gabriel Englander, Patrick Hunnicutt

**Affiliations:** ^a^Bren School of Environmental Science and Management, University of California, Santa Barbara, CA 93117; ^b^Development Research Group, The World Bank, Washington, DC 20433; ^c^Environmental Science and Policy, Chapman University, Orange, CA 92866; ^d^School of Planning, Public Policy, and Management, University of Oregon, Eugene, OR 97403

**Keywords:** protected areas, private sector management, wildlife conservation, armed conflict, economic development

## Abstract

Protected areas can conserve wildlife and benefit people when managed effectively. African governments increasingly delegate the management of protected areas to private, nongovernmental organizations, hoping that private organizations’ significant resources and technical capacities actualize protected areas’ potential. Does private sector management improve outcomes compared to a counterfactual of government management? We leverage the transfer of management authority from governments to African Parks (AP)—the largest private manager of protected areas in Africa—to show that private management significantly improves wildlife outcomes via reduced elephant poaching and increased bird abundances. Our results also suggest that AP’s management augments tourism, while the effect on rural wealth is inconclusive. However, AP’s management increases the risk of armed groups targeting civilians, which could be an unintended outcome of AP’s improved monitoring and enforcement systems. These findings reveal an intricate interplay between conservation, economic development, and security under privately managed protected areas in Africa.

## Significance

Mitigating the global biodiversity crisis requires a significant expansion in effectively managed protected areas. Private nongovernmental organizations may facilitate this expansion by managing protected areas on governments’ behalf. Our quasi-experimental approach focusing on protected areas in Africa shows that private management substantially benefits wildlife populations and augments tourism. However, private management’s impacts on rural wealth are inconclusive, and we find some evidence that private management undermines the physical security of communities living near protected areas. Strengthening local communities’ involvement in private protected area management may help realize protected areas’ full potential benefits for both wildlife and people.

Our planet is experiencing a biodiversity crisis. Anthropogenic threats including land use change, overfishing and overhunting, pollution, and climate change are causing large-scale reductions in plant and animal populations (1–4). Such losses can threaten human health (5–7), slow economic development (8, 9), and deepen inequality (10).

The international community has responded to this crisis by advocating for the expansion and enhancement of protected areas. A key development in these efforts occurred recently in 2022 when 196 countries ratified the “Kunming-Montreal Global Biodiversity Framework” (GBF) (11). This framework sets ambitious targets for biodiversity conservation, most notably to cover 30% of the world’s terrestrial, marine, and freshwater ecosystems with effectively managed protected areas by 2030 (12). The GBF’s focus on protected areas is supported by research demonstrating their potential to deliver benefits to both biodiversity and people: protected areas can conserve plant and animal populations by reducing habitat loss and hunting (13–16), promote rural economic development (17, 18), and aid in adaptation to climate change (19).

However, despite appreciation for their importance, and goals to expand them, many protected areas are failing to realize their potential. Security challenges, inadequate financial resources, limited technical capacity, and inequitable governance are hindering protected area management (13, 20–27).

In response to these challenges, African governments are increasingly turning to private nongovernmental organizations (NGOs) for assistance (28–31). Under collaborative management models, African governments partner with or fully delegate control over protected area management to NGOs (32–34). NGOs may offer advantages such as greater access to donor funding, technical expertise, and reduced susceptibility to corruption (29, 34). However, concerns arise regarding their legitimacy and potential adoption of militarized, “fortress-style” conservation methods (35). Such methods could exacerbate political violence and perpetuate exclusionary colonial-era conservation practices (24–27, 36). Despite these potential trade-offs, comprehensive evaluations of private sector involvement in protected area management are scarce (37).

Here, we use a quasi-experimental approach to evaluate the impacts of private sector protected area management on people and wildlife in Africa. We employ the case of African Parks (AP), a South Africa-based nonprofit NGO that partners with African governments to manage protected areas. AP’s primary mission is to conserve, restore, and connect wildlife populations across regional landscapes in Africa (38). The organization’s scope is continental, and its interventions are ambitious. For example, AP often reintroduces large mammals, sometimes in unprecedented numbers, to protected areas where they were historically lost due to overhunting (38). Many of the species AP works to restore are threatened or endangered, such as African lions, African wild dogs, rhinoceroses, and elephants. Such large-scale conservation and reintroduction projects have the potential to not only benefit species of high conservation concern but also, through reinstating the ecological roles of large-bodied animals, restore ecosystems broadly (39–41).

Wildlife conservation via law enforcement lies at the center of AP’s management model (38, 42). Indeed, AP’s website states the “most critical and foundational component for the long-term sustainability of any park is effective protection,” which it considers to be its “top priority” (43). As such, the organization often leverages its considerable financial resources to employ heavily armed park rangers and equip them with helicopters, light aircraft, and other monitoring and enforcement technologies (38). The militarized style of conservation AP pursues perhaps reflects the conflict-affected settings in which it operates. For example, AP rangers active across Central Africa sometimes confront armed groups who hunt wildlife and extract natural resources from within park boundaries (44–46).

At the same time, AP seeks to maximize the benefits of protected areas for local people by creating job opportunities, constructing and financing infrastructure, schools, and health clinics, offering scholarships for local students, and promoting tourism. As these programs suggest, AP views healthy wildlife populations, effectively managed protected areas, and economic development as inextricably linked (38, 42).

While several NGOs manage protected areas in Africa (e.g. the Wildlife Conservation Society, the Virunga Foundation) (34), AP currently manages more land and protected areas in Africa than any other NGO: over 200,000 km^2^ across 22 protected areas in 12 different countries. Even so, AP aims to expand the number of protected areas under its management to 30 protected areas by 2030, and potentially to over 90 protected areas in the long term (38).

Due to its focus on restoration, AP often seeks out historically underfunded and ineffectively managed protected areas that have experienced substantial wildlife declines and local extinctions (38, 42). AP comes to manage protected areas through mandates it establishes with national governments. In the past, both AP and national governments have initiated discussions to form these mandates (42, 47, 48). Discussions are private, and mandates are both expansive and long-term, granting AP complete authority to manage and govern protected areas, including processes related to hiring, revenue generation, and security provision. AP is accountable to the objectives established in mandates, and either party can withdraw should circumstances change such that the partnership is no longer viable (47, 48). Mandates currently average 20 y (38).

Studying AP management offers valuable insights into real-world impacts of private sector involvement in conservation in Africa. AP’s ambitious vision and its success in acquiring numerous and expansive long-term mandates positions it to be an influential force in wildlife conservation in Africa for the foreseeable future. At the same time, the organization’s management strategies include potential trade-offs which broadly characterize the dilemma of private sector protected area management.

The primary objective of this paper is to estimate how AP management impacts wildlife, socioeconomic, and security-related outcomes relative to a counterfactual scenario in which the organization’s protected areas remained under government management. To estimate the effects of AP management, we rely on two key features of our setting: 1) the staggered timing of protected areas being transferred to AP management; 2) a set of control protected areas that AP has identified as candidates for future management given their similarities with protected areas already in AP’s portfolio.

**Fig. 1.**
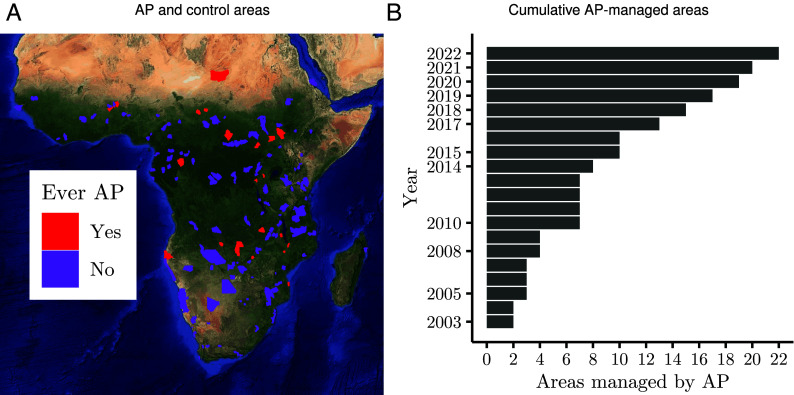
Research design compares changes in outcomes among protected areas transferred to African Parks (AP) management to changes in outcomes among similar areas that have never been managed by AP. (*A*) Protected areas ever managed by AP are filled red, and control group areas that are managed by governments and that have never been managed by AP are filled purple. Control areas are those determined by AP as meeting their criteria for future management. (*B*) Number of protected areas managed by AP by year.

The governments of 12 African countries transferred management of 22 protected areas to AP between 2003 and 2022 (Fig. 1*B*). These countries are Angola, Benin, Central African Republic, Chad, Republic of Congo, Democratic Republic of the Congo, Malawi, Mozambique, Rwanda, South Sudan, Zambia, and Zimbabwe. We implement a recently developed dynamic difference-in-differences estimator to reveal how the transference of these 22 protected areas to AP affects both wildlife and people. The estimator expands upon the canonical difference-in-differences approach by accounting for the staggered onset of treatment across units (49). Ultimately, we compare the before-after change in an outcome in protected areas transferred to AP management to the concurrent change in an outcome in protected areas always managed by governments.

One substantial challenge for any evaluation of protected area management is identifying a valid counterfactual, as different protected area management systems are not randomly assigned (37, 50). We mimic AP’s treatment assignment process to overcome this challenge, forming our control group from protected areas AP recently identified as ideal candidates for future management (51). AP selected these protected areas, referred to as “anchor areas,” because they share key characteristics with the protected areas currently managed by AP. Specifically, anchor areas are 1) extensive landscapes (exceeding 500 km^2^), 2) very likely to have a strong legal status (e.g., national park designation), 3) experience limited agricultural activity within their boundaries, and 4) contain the presence or potential to sustain significant wildlife populations, particularly those of large mammals. Anchor areas under private management were removed from our sample—given our goal of evaluating the impacts of transferring protected area management to private entities—leading to a final control group of 123 government-managed protected areas (Fig. 1*A*). We believe this process for constructing our control group strengthens our ability to identify changes in outcomes that are due to AP management, compared to an approach where the control group includes all non-AP-managed protected areas in Africa.

## 1. Results

**Fig. 2.**
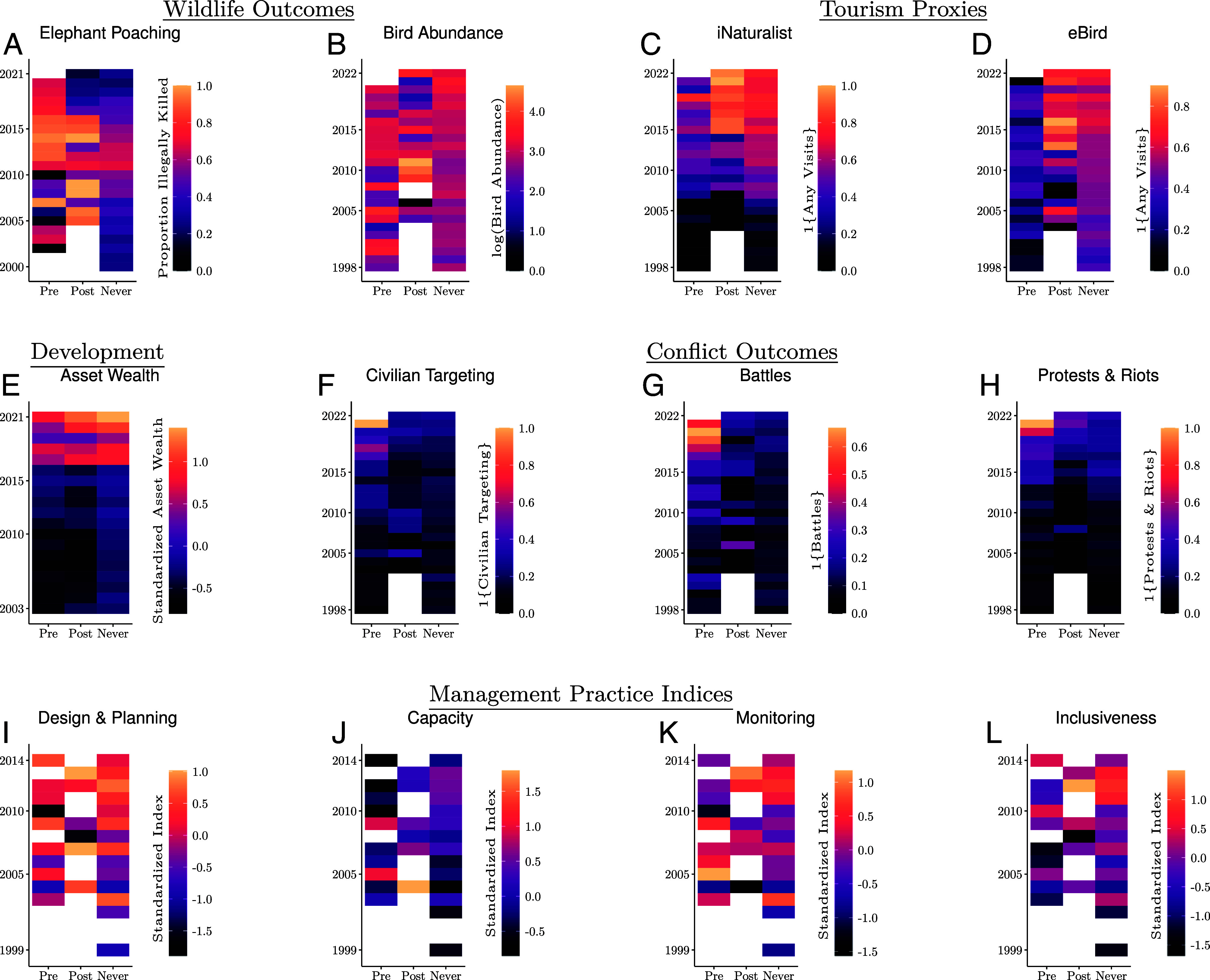
Annual mean outcomes and management indices in protected areas prior to AP management (Pre), after AP management (Post), and in protected areas always managed by governments (Never). Data from (*A*) Monitoring the Illegal Killing of Elephants, (*B*) eBird, (*C*) iNaturalist, (*D*) eBird, (*E*) Atlas AI, (*F*–*H*) Armed Conflict Location and Event Database, and (*I*–*L*) Management Effectiveness Tracking Tool. Each cell in the heatmap is the average value of the dependent variable for a specific group (x-axis) in a given calendar year (y-axis). Blank (white) cells indicate no data or protected areas in a group that year.

To evaluate outcomes between AP and government management, we leverage large-scale datasets on wildlife, asset wealth, conflict, and management practices (52). This comprehensive approach extends prior research documenting the effects of protected areas on land use change (53–55). Fig. 2 displays annual mean values from these diverse datasets in AP-managed protected areas before and after their transference to AP, and in government-managed protected areas. Stark differences in some outcomes pre- and posttransference to AP (Fig. 2*A*, *C*, and *D*) underscore the rich variation in our data, forming a foundation for a more careful analysis. However, these descriptive statistics are not indicative of AP management effects. Our subsequent difference-in-differences analysis, normalizing time relative to transference and controlling for confounding factors, is essential to accurately attribute changes to AP management.

To assess the validity of our difference-in-differences research design, we begin by statistically comparing protected areas managed by AP to our control group of protected areas in terms of variables unaffected by protected area management and in terms of outcomes prior to AP management. Protected areas managed by AP do not statistically differ from the control group in terms of area (km^2^), longitude, latitude, or annual precipitation (*SI Appendix*, Table S1). However, AP-managed areas experience more extreme heat. They also exhibit uniformly worse preperiod outcomes, though not all differences are statistically significant (*SI Appendix*, Table S2). Prior to transference, areas that will go on to be managed by AP experience higher elephant poaching, lower bird abundances, less tourism, more armed conflict, lower asset wealth, and less effective management practices. Our examination of outcomes in each of the 5 y preceding transference confirms the intuition that governments may be transferring protected areas where conditions are difficult and deteriorating. Across all but one of the variables, outcomes are similar or worsening in the protected areas that will be transferred to AP management, compared to protected areas that will continue to be managed by governments (*SI Appendix*, sections A and B).

It is not surprising that protected areas in AP’s portfolio fare worse in terms of their preperiod outcomes than do protected areas in the control group. AP’s task is a difficult one, as it purposefully seeks protected areas that governments have historically struggled to manage. Governments may also be inclined to transfer management of their most challenging protected areas, given how the prospect of additional resources motivates the broader shift toward private protected area management in Africa (29). However, the differences between the two groups of protected areas largely do not undermine our ability to infer the effects of AP management. The outcomes we examine are unlikely to benefit from mean reversion (improvements that would have occurred by themselves). Therefore, we can explicitly characterize the bias the preperiod differences we estimate may induce. Any improvements we detect due to AP management may be underestimates, and any worsening in outcomes may be overestimates. We also urge readers to consider how our control group strengthens our research design. Using AP’s selection process to identify the counterfactual to AP management balances concerns about internal and external validity, relative to a research design where we omit from our control group all areas that are dissimilar (56). This decision supports both our narrow goal of evaluating AP’s impacts and our broader goal of estimating the trade-offs of transferring any government-managed protected area to a private entity, not just particularly successful or struggling government-managed protected areas.

### 1.1. Wildlife Outcomes.

Biodiversity conservation is the foremost goal of protected areas, as well as of AP itself, rendering wildlife outcomes a primary gauge of the efficacy by which AP manages parks. Our evaluation requires wildlife data that meet two criteria. First, the data must be collected in a consistent manner, or else contain information regarding surveyor effort that can be used to make observations comparable across different areas and time periods. Second, they must provide sufficient spatial and temporal coverage to facilitate the application of the dynamic difference-in-differences estimator. Only two datasets meet these requirements: Monitoring the Illegal Killing of Elephants, which measures elephant poaching, and eBird, which provides data regarding bird abundances (57, 58).

**Fig. 3.**
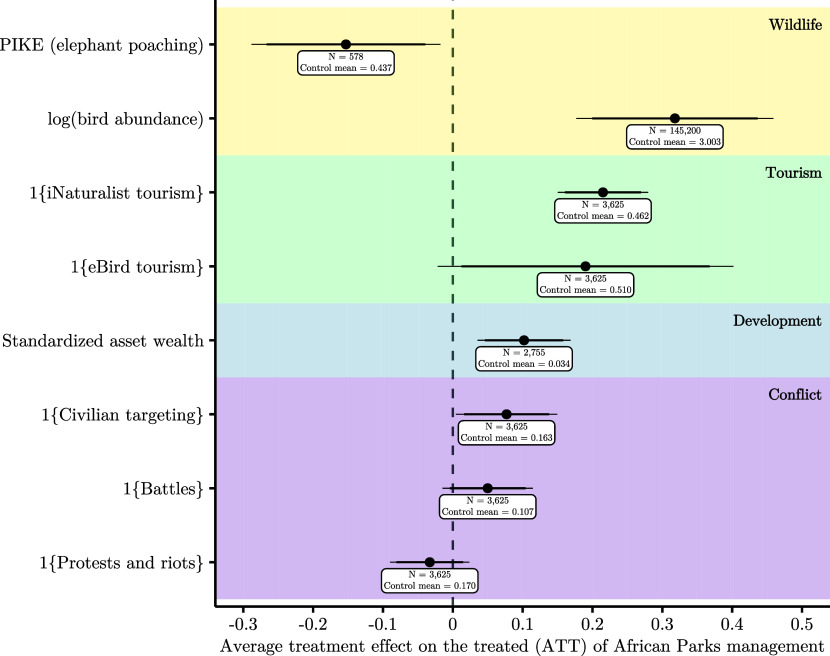
Average effect of AP management on wildlife, tourism, economic development, and conflict. Each row presents the result of a separate regression. The y-axis specifies the dependent variable in each regression. The points display the Average Treatment effect on the Treated (ATT), the average effect of AP management on a given dependent variable. The thick and thin bars represent the 90% and 95% CIs, respectively. SEs are clustered at the protected area level for each regression. The text boxes display the number of observations and the mean of the dependent variable among control group protected areas. *SI Appendix*, Table S3 presents these results in numeric format.

We estimate that AP management reduces elephant poaching by a statistically significant 15.3 percentage points, which equates to a 35% reduction in elephant poaching relative to the mean poaching rate among control areas (Fig. 3 and *SI Appendix*, Table S3, Row 1). Areas destined for AP management experience rising rates of elephant poaching over the 5 y before transference, which suggests that law enforcement is weakening or poaching effort is escalating (*SI Appendix*, Fig. S1). AP may reduce elephant poaching by even more than 35% because in the absence of AP management, elephant poaching would likely have continued to increase (*SI Appendix*, section A.1). Spillover reductions in elephant poaching near areas transferred to AP management provide further evidence that the true reduction in elephant poaching due to AP management may be even larger than 35% (*SI Appendix*, section A.1.1).

In our evaluation of the effect of AP on bird abundances, we replicate the primary specification of a recent paper that used eBird data to study the relationship between air pollution regulation and bird abundances (59). This approach limits researcher degrees of freedom (*SI Appendix*, section A.2). We estimate that AP management significantly increases bird abundances by 0.318 log points, or approximately 37% (Fig. 3, Row 2). The downward trend in bird abundances prior to AP management means we may underestimate the true increase in bird populations (*SI Appendix*, Fig. S2).

This increase in bird abundances may occur because AP reduces bird hunting. Plausible alternatives do not fully explain our findings, such as AP changing where or when birder observations occur within protected areas (*SI Appendix*, Figs. S3 and S4), or AP changing the composition of birders toward those who are more skilled or more likely to report observing greater numbers of birds (*SI Appendix*, Fig. S5). Our additional replication of a flexible method of controlling for surveyor effort lends further credence to these results (*SI Appendix*, Fig. S6). We find similar year-by-year changes when we estimate the effect of AP management on the number of bird species observed, though the average effect is slightly negative in this case (*SI Appendix*, Fig. S7 and Table S4). The marked rise in bird abundances following transference to AP management reinforces the notion that AP management improves wildlife outcomes.

### 1.2. Tourism.

We now turn our attention to the effect of AP management on tourism. There are several reasons why AP might boost tourism. The increased wildlife populations under AP management could attract more tourists, or AP’s potentially superior ability to market its parks internationally compared to government-managed parks could increase visitation. Due to the lack of comprehensive data on actual tourist visits to parks across Africa, we rely on the following proxies as the best available measures of tourism.

We first utilize data from the widely used citizen science platform iNaturalist to approximate tourism visits (60), following prior research leveraging photographs of wildlife posted to social media platforms like Flickr to measure tourism (61, 62). Users of iNaturalist upload geolocated and timestamped photos of flora and fauna, providing information regarding the location and timing of park visits (*SI Appendix*, section A.3). It is important to note, however, that we cannot use iNaturalist data as a measure of wildlife outcomes due to the absence of information on surveyor effort.

We estimate that AP management significantly increases the probability of positive iNaturalist visits by 21.5 percentage points, or by 47% relative to the mean among control areas (Fig. 3, Row 3 and *SI Appendix*, Fig. S8). “Positive visits” refers to the presence of any iNaturalist observations in a given protected area-year; this condition is met slightly less than half of the time in the control group. We obtain similar results when we exclude observations submitted by potential protected area staff (*SI Appendix*, Fig. S9).

To supplement this finding, we also use eBird data as a proxy for tourism (*SI Appendix*, section A.4). On average, AP management increases the probability of positive eBird visits by 19 percentage points, or by 37% relative to the mean among control areas (Fig. 3, Row 4).

For both tourism proxies, interpretation of these effects is not complicated because there is no trend in preperiod outcomes (*SI Appendix*, Figs. S8 and S10). We also obtain positive effects when we use the log number of iNaturalist or eBird visits as the dependent variable (*SI Appendix*, Fig. S11). Considering the two proxies together, it seems likely that AP management increases tourism.

### 1.3. Economic Development.

In addition to its efforts to conserve wildlife and stimulate tourism, AP initiates local economic development projects in communities adjacent to the protected areas they manage. We use data on “asset wealth” from Atlas AI, a private data provider, to test whether AP management enhances local economic well-being. Atlas AI uses daytime and nighttime optical imagery to predict asset wealth as measured in the demographic and health surveys (DHS) Program (63). After training a machine learning model on DHS data, Atlas AI predicts asset wealth for the continent of Africa. The data are produced at an annual frequency and delineated by second-level administrative divisions, spanning the years from 2003 to 2021. We filter the data to include only those administrative divisions that are located within a 25 km radius of our protected areas (*SI Appendix*, Fig. S12*A*).

The average effect of AP management on asset wealth is 0.102 SDs, with a SE of 0.034 (Fig. 3, Row 5). However, we cannot interpret this increase in economic well-being as being solely attributable to AP management due to elevated levels of asset wealth immediately prior to transference (*SI Appendix*, Fig. S12*B*). This suggests that communities near protected areas destined for AP management are already becoming richer at a faster rate than communities near protected areas that never come under AP management. The posttransference stability of asset wealth could, therefore, be a continuation of this preexisting upward trend rather than a consequence of AP’s actions.

### 1.4. Conflict.

Finally, we investigate whether AP’s activities affect conflict within and around the protected areas it manages. AP’s militarized law enforcement components might generate positive spillover effects, deterring crime and forms of political violence linked to the extraction of natural resources (64–66). However, it is also possible that AP’s law enforcement exacerbates local insecurity. For example, if AP undermines an armed group’s revenue generation by blocking their access to protected areas with valuable natural resources, then that armed group may be more likely to target civilians as a form of revenue generation (67, 68). Indeed, Section 1502 of the Dodd-Frank Act—a de facto prohibition on US manufacturers’ sourcing of tin, tantalum, and tungsten from the Democratic Republic of the Congo—undercut local armed groups’ profits but increased the looting of civilians and violent clashes over mining territories (69, 70). Alternatively, AP management may trigger protests if it both limits local communities’ access to the resources within protected areas and fails to provide local communities with alternative sources of economic opportunity, similar to the local effects of mining concessions (71).

Accordingly, we use the Armed Conflict Location and Event Database (72) to measure the presence of three forms of conflict in and around the protected areas in our sample: violence against civilians (“civilian targeting”), battles, and protests and riots. We define our spatial unit of observation as the area within a protected area’s boundaries plus a 25-km buffer around the protected area’s boundaries. We include these buffer zones in our analysis to capture possible spillover in AP management’s effect on conflict. We use relatively small buffers—in comparison to research investigating the spillover effects of climatic shocks on conflict (73)—because we focus on local security conditions. Given the scale of the mechanisms described above, we hesitate to attribute distant changes in conflict to AP management. *SI Appendix*, section A.6.2 discusses this decision in greater detail and reports a robustness check where we recompile our results using smaller and larger buffers (*SI Appendix*, Fig. S18).

We find suggestive and concerning evidence that AP makes civilian targeting more likely in and around the protected areas it comes to manage (Fig. 3, Row 6). The probability of any civilian targeting occurring in AP-managed protected areas increases by 7.7 percentage points posttransference. This estimate represents a 47.2% increase in the presence of civilian targeting relative to the control mean, and in the 5 y before transference, the presence of civilian targeting is similar in protected areas that will be transferred to AP and in those that will not be (*SI Appendix*, Fig. S13*A*). As in our tourism analysis, we prefer binary measures of conflict in order to reduce potential measurement error stemming from reporting bias (*SI Appendix*, section A.6 and Fig. S15). However, it is important to note that we do not find an effect of AP management on the number of civilian targeting events (*SI Appendix*, Fig. S16). We also find no clear evidence that AP changes the probability of any battles occurring within 25 km of the protected areas it manages, nor does AP appear to affect the probability of any protests and riots within 25 km of the protected areas it manages (Fig. 3, Rows 7 to 8).

### 1.5. Mechanisms.

Which aspects of AP management might explain its capacity to improve wildlife conservation and tourism but exacerbate one form of conflict? We utilize survey data on management practices recorded with the Management Effectiveness Tracking Tool (METT) (74). METT is a standardized questionnaire that is typically filled out as a group exercise among protected area managers and other stakeholders (75, 76). It is designed to characterize the management and governance of protected areas by quantifying aspects such as planning, resource levels, law enforcement, and stakeholder involvement. These data are self-reported and only available for some protected areas and years; nonetheless, they represent the best opportunity to quantitatively understand the ways in which AP management differs from government management of protected areas.

Following previous research, we group responses to the METT’s 30 questions into four distinct categories (53, 76):

**Design and planning:** This category captures the legal framework of the protected area and whether its strategic design and planning promote effective operations (77). AP management increases this dimension by 0.683 SDs, reflecting AP’s proactive and robust planning approach, although this effect is not statistically significant due to the limited METT data available (Table 1).
**Capacity and resources:** This dimension relates to the availability and management of resources, including staff count and budget. Effective management requires adequate resources and capacities, encompassing well-trained staff and sufficient equipment to enforce regulations, diminish threats, and enhance ecological conditions (76, 77). We find an increase of 0.581 SDs in this category due to AP management, signifying AP’s effective fundraising and resource management (Table 1). However, this effect is also not statistically significant.
**Monitoring and enforcement systems:** This category assesses the enforcement capacity of the protected area, evaluates whether its legal framework permits action against the protected area’s primary threats, and measures understanding of the biological conditions within the protected area. AP management significantly improves this dimension, with an increase of 0.926 SDs (Table 1). This result aligns with AP’s focus on law enforcement and monitoring.
**Decision-making inclusiveness:** This dimension pertains to stakeholder involvement and their influence on management decisions. Including diverse stakeholders can improve the perceived legitimacy of the protected area and facilitate its congruence with local social and ecological contexts (78). We find that AP management reduces decision-making inclusiveness by 0.292 SDs, though the effect is not statistically significant (Table 1). This decrease suggests that AP’s centralized governance reduces stakeholder involvement in decision-making.

**Table 1.** Average effect of AP on management indices

Dependent variable	Coefficient	SE	*N*	Control mean
(1)	(2)	(3)	(4)	(5)
Design and planning	0.683	(0.487)	154	0.044
Capacity and resources	0.581	(0.618)	155	−0.005
Monitoring and enforcement systems	0.926	(0.280)	155	0.013
Decision-making inclusiveness	−0.292	(0.358)	153	0.037

Each row presents the result of a separate regression. Column 1 specifies the dependent variable in each regression. Column 2 reports the regression coefficient corresponding to AP’s effect; it is identified from the before-after change in a given management index in protected areas transferred to AP, compared to the concurrent change in the management index in protected areas always managed by governments. Column 3 displays the Column 2 coefficient’s SE. Column 4 reports the number of observations in the regression and Column 5 shows the mean of the dependent variable among control group protected areas.

## 2. Discussion

The trend in Africa toward private management of protected areas, exemplified by AP, reflects key themes in broader discussions regarding the privatization of public services (79). Related studies examine the benefits and drawbacks of privatization in diverse areas, from healthcare to transportation (80, 81). Our analysis extends this debate, offering insights into when and why private management might be effective in the field of environmental conservation. Our findings invite further investigation into whether AP’s successes can be replicated by other organizations outside of Africa.

We find that AP management improves outcomes for wildlife, likely due to the organization’s ability to translate its considerable financial resources into expanded and sophisticated monitoring and enforcement activities. While our results pertain specifically to elephants and birds, we suspect AP management benefits other wildlife species too, especially medium- to large-bodied species, many of which are threatened by overhunting in Africa (82, 83). That AP can improve outcomes for wildlife in active conflict zones, where wildlife can be especially prone to overhunting (84), is both remarkable and speaks to the enormous potential of private protected area management to conserve wildlife in Africa.

AP’s impact on local conflict dynamics, however, raises serious ethical and strategic concerns about private sector stewardship of protected areas. While AP’s intensified antipoaching strategies may better protect wildlife and bolster the security of its rangers, they may also inadvertently trigger the targeting of civilians by armed groups. Such dynamics align with the notion that increasing the regulation of natural resources that armed groups rely on for revenue generation can erode political stability (*SI Appendix*, section A.6.1 and Fig. S17). Rebel groups with extensive resource endowments are capable of mounting complex attacks on vulnerable targets (85), and government forces also threaten civilians’ safety in resource-rich regions (86). The subset of AP-managed protected areas in active conflict zones, such as Garamba National Park in the Democratic Republic of Congo and Pendjari and W National Parks in Benin, likely are driving the increase in civilian targeting we estimate (*SI Appendix*, Fig. S14).

Our findings principally underscore the need to strengthen local communities’ involvement in protected area management. Recall that we find suggestive evidence of transference to AP coinciding with lower levels of decision-making inclusiveness in protected area management. If increased insecurity is one cost of transferring protected areas to private organizations, then the normative argument for bridging the gap between communities’ safety and private organizations’ conservation activities is even stronger. Doing so also may have instrumental value: higher levels of insecurity could undermine communities’ perceptions of protected area management, reducing rangers’ ability to gather information critical to antipoaching efforts (87). Forming what some call “inclusive” antipoaching units—whereby rangers are accountable to local communities instead of external organizations—may help safeguard wildlife without placing nearby communities’ safety at risk (88, 89).

Yet our analysis of AP’s effect on economic development provides some optimism that privately managed protected areas can benefit people and wildlife. For example, we find that tourism increases in protected areas after they are transferred to AP. This hints at the economic potential of private sector involvement in protected area management. Of course, our results rely on tourism proxy data, and actual visitor numbers should be obtained to confirm these findings. Moreover, the distribution of tourism benefits within local communities remains unknown, warranting future exploration.

It also appears that AP’s positive conservation impact does not impair economic development, in contrast to the traditional view that conservation and development are competing objectives (90). This trade-off between conservation and economic development may be occurring prior to AP management, as indicated by the upward pretransference trend in asset wealth (indicating economic development) concurrent with the downward pretransference trend in elephant poaching (indicating reduced conservation). However, asset wealth remains stable and elevated in protected areas following their transference to AP, even as elephant poaching rates decline substantially. It is important to emphasize that the absence of evidence of an economic development cost does not imply that AP management increases economic development; the upward pretransference trend in asset wealth precludes that inference.

Finally, our results highlight a continued need for careful evaluations of privately managed protected areas, particularly regarding their impacts on nearby communities. People living near protected areas have much to gain or lose from private management, yet their voices are seldom captured in the observational data used to gauge protected area effectiveness. Large field-based data collection projects enabling the careful measurement of local peoples’ experiences of protected area management will be critical for future research. For example, such data would provide greater certainty in determining whether the increase in tourism we estimate above benefits communities surrounding AP’s protected areas. These data may also enable tests of how different forms of protected area management shape human-wildlife conflict, considering the positive outcomes for wildlife that we observe.

We show that transferring protected areas to private entities can address some of the challenges undermining effective protected area management in Africa, supporting global initiatives to safeguard Earth’s biodiversity. However, our study also suggests private protected area management is not a panacea. AP management specifically appears to have unintended effects on local security conditions via its monitoring and enforcement activities, and it is concerning that we find suggestive evidence of AP management reducing decision-making inclusiveness. Addressing these shortcomings will be critical for ensuring that protected areas in Africa achieve their full potential, not only for the continent’s wildlife but also for its people.

## 3. Materials and Methods

We implement a recently developed dynamic difference-in-differences estimator to reveal how management by AP compares to management by governments (49). This estimator compares the before-and-after change in an outcome among protected areas transferred to AP management with the contemporaneous change in outcomes among areas always managed by governments. It improves upon the traditional “two-way fixed effects” estimator by avoiding “forbidden comparisons,” which occur when units treated in earlier years of the study period are used as control units in estimating effects on units treated in later years (91). In our context, this means excluding from the control group those cohorts of protected areas transferred to AP management in earlier years when assessing the impact on areas transferred later. Here, “cohorts” refer to groups of protected areas transferred to AP in specific calendar years (e.g., the two protected areas transferred to AP in 2003 represent one cohort). Avoiding forbidden comparisons is crucial, as they can lead to paradoxical estimates, such as an average treatment effect that has the opposite sign of the individual treatment effects it is composed of (92, 93).

The dynamic estimator we implement analyzes the impact of AP management at multiple year-long time periods relative to the date of transference, such that trends can be established through time and changes more clearly attributed to AP management. This analysis spans from 5 y before transference to 10 y posttransference. We bottom code (set a lower limit for) the leads at 6 y before and top code (set an upper limit for) the lags at 11 y after transference. We exclude the bottom and top coded coefficients when we display the regression results in figures as these coefficients do not have a clear interpretation.

The first step of our adopted approach estimates, via ordinary least squares regression, a linear two-way fixed effects model that interacts relative time period indicators (ℓ) with cohort indicators (e):

[1] $$\begin{array}{c} Y_{\mathrm{it}}=\alpha_{i}+\lambda_{t}+\sum_{e}\sum_{\begin{array}{c} \ell =-6\\ \ell \neq -1 \end{array}}^{11}\delta_{e\ell }\left(1\left\{E_{i}=e\right\}\cdot D_{\mathrm{it}}^{\ell }\right)+\gamma X_{\mathrm{it}}+\epsilon_{\mathrm{it}}, \end{array}$$

where $Y_{\mathrm{it}}$ is an outcome in protected area i in calendar year t, $\alpha_{i}$ are the protected area fixed effects (binary indicator variables for each protected area), $\lambda_{t}$ are the calendar year fixed effects, $\delta_{e\ell }$ represents the treatment effect for cohort e in relative period ℓ, $1\{E_{i}=e\}$ is a binary indicator that equals 1 if protected area i belongs to cohort e, $D_{\mathrm{it}}^{\ell }$ is a binary indicator that equals 1 if protected area i is ℓ years away from transference to AP management, $X_{\mathrm{it}}$ is a matrix of weather control variables (defined below), and $\epsilon_{\mathrm{it}}$ is the error term (49). The indicator $D_{\mathrm{it}}^{\ell }$ always equals 0 for protected areas always managed by governments. This regression avoids forbidden comparisons by estimating a separate effect for every cohort and relative period combination. The regression omits the indicator for the year immediately preceding transference (ℓ=−1) to avoid multicollinearity. The protected area fixed effects control for all time-invariant characteristics of each protected area, such as location and inherent habitat features, while the calendar year fixed effects account for time-varying factors that affect all protected areas uniformly, such as global economic trends and global demand for elephant ivory.

The second step estimates weights for the treatment effect coefficients ($\delta_{e\ell }$) based on the proportion of observations each cohort represents in each relative period. The final step computes the weighted average treatment effect for each relative period. We use the commands feols and sunab from the R package fixest to perform the estimation procedure (94). We present all relative period estimates in *SI Appendix* figures. The Average Treatment effect on the Treated estimates shown in the main text tables are the weighted averages over the posttransference relative treatment period coefficients (0≤ℓ≤10), where the weights for each relative period coefficient are based on the proportion of the treated group (areas managed by AP) in the overall population during that specific relative period (49).

Our control group comprises protected areas that AP has determined meet their established criteria for potential future management, which AP refers to as “anchor areas.” Polygons demarcating the boundaries of AP’s anchor areas are publicly available (51). We identify individual protected areas in our control group in three steps. First, we used Quantum Geographic Information System (QGIS) to manually select all polygons from each of the World Database of Protected Areas’ (WDPA) shapefiles that overlapped with the boundaries of AP’s anchor areas. Second, we eliminated duplicate entries from the selected WDPA boundaries. Finally, we manually validated the remaining WDPA boundaries to confirm that protected areas in our control group match the set of polygons displayed on AP’s map of anchor areas. In three cases, we used QGIS and georeferenced polygons from either United Nations Educational, Scientific and Cultural Organization (UNESCO) or the literature (95, 96) to manually create shapefiles for AP anchor areas that did not have shapefiles in the WDPA.

Several protected areas in our control group are partly managed by governments and partly managed by an NGO other than AP. We retain these protected areas in our control group because they do not employ a “delegated” management model like AP, where the NGO has full control over management decisions (29). Several of AP’s anchor areas are privately managed, so we removed these from our control group. To determine which areas were privately managed, we first reviewed the literature for mention of “delegated” or “collaborative” management models. We then exhaustively reviewed the websites of the following major conservation organizations that support protected area management in Africa: Wildlife Conservation Society, World Wildlife Fund, Frankfurt Zoological Society, Zoological Society of London, Peace Parks Foundation, Born Free Foundation, and African Wildlife Foundation. We then searched the web one anchor area at a time to look for any language that suggested the area might be privately managed.

We exclude two protected areas from the control group because they are in AP’s incubator program. These areas are being managed by a different NGO, but AP is providing advice and training. We display the full list of control and treatment-protected areas in *SI Appendix*, Table S5. We also exclude three areas that were briefly managed by AP before their withdrawal to reallocate resources elsewhere, at least temporarily. Our final control group includes 123 protected areas.

The validity of our estimates relies most importantly on the “parallel trends” assumption: the change in outcome in control protected areas represents the change in outcome that would have happened in treatment areas if management of those areas was not transferred to AP (91). We consider the validity of the parallel trends assumption for each outcome separately based on the levels and trend in the relative period estimates in the years prior to transference. When trends in preperiod coefficients exist, we use context-specific knowledge to characterize the likely direction of the bias (97, 98). Related to the parallel trends assumption is the “no anticipation” assumption, that AP management has no causal effects on outcomes prior to transference (91). We believe the no anticipation assumption is likely to be satisfied because negotiations between AP and country governments prior to transference are not publicly disclosed.

Unless otherwise noted, the data in all regressions are at the level of protected area-year, and the control variables are protected area fixed effects, calendar year fixed effects, and functions of temperature and precipitation. Controlling for weather may improve the precision of estimated effects on outcomes that depend on weather, as well as avoid omitted variables bias if AP management incidence or transference timing depends on contemporaneous weather. We cluster SEs at the protected area level because that is the level at which treatment is assigned (99).

The temperature and precipitation control variables originate from the ERA5-Land dataset (100). The temporal resolution of the dataset is hourly, and the spatial resolution is approximately 9 by 9 km grid cells. We calculate nonlinear transformations of temperature and precipitation at the original resolution of the data before aggregating to the level of protected area-year. Specifically, we calculate squared and cubed precipitation in meters, and degree hours in 3 ^°^C bins. For example, an observation with a temperature of 13 ^°^C would have a value of 2 in the 11-to-14 ^°^C bin (because 13 min 11 is 2) and a value of 0 in all other bins. We convert from degree hours to degree days and consolidate some of the sparse degree day bins. The weather control variables we include in our regressions are a third-order polynomial in precipitation in m, and the following 12 degree day bins: −19 to 5 ^°^C, 5 to 8 ^°^C, 8 to 11 ^°^C, 11 to 14 ^°^C, 14 to 17 ^°^C, 17 to 20 ^°^C, 20 to 23 ^°^C, 23 to 26 ^°^C, 26 to 29 ^°^C, 29 to 32 ^°^C, 32 to 35 ^°^C, and 35 to 41 ^°^C. Controlling for degree days rather than temperature polynomials better accounts for the effect of temperature on agricultural yields, which is important because agricultural yields could have direct effects on some of our outcome variables, such as asset wealth and those related to conflict (101).

In addition to calculating weather control variables for each protected area-year, we follow the same procedure to calculate the same weather control variables for each protected area’s year and 25 km, 50 km, and 75 buffers. We use this second set of weather control variables in the primary asset wealth and conflict regressions because those regressions include data inside and within 25km of protected areas. We use the 50 km and 75 km weather control variables in *SI Appendix*, Fig. S18.

We detail all outcome variables and specific regressions in *SI Appendix*.

During the writing of the manuscript, we used GPT-4 in order to draft and revise text. After using this tool, we reviewed and edited the content as needed and take full responsibility for the content of the publication.

## Supplementary Material

Appendix 01 (PDF)

## Data Availability

All data are available in figshare at https://doi.org/10.6084/m9.figshare.25560351 (52). The only exception is the raw asset wealth data, obtainable directly from Atlas AI. However, we provide in figshare the asset wealth values for each protected area and year in our study, along with the code used to derive these values from the raw data.
