# Supplementary material for: Private management of African protected areas improves wildlife and tourism outcomes but with security concerns in conflict regions
Source: Proc Natl Acad Sci U S A. 2024 Jul 1;121(29):e2401814121. doi: 10.1073/pnas.2401814121 (PMC11260162; doi:10.1073/pnas.2401814121)
Supplement: Supplementary file 1 — Appendix 01 (PDF) [file pnas.2401814121.sapp.pdf]

PNAS

Supporting Information for

Private management of African protected areas improves wildlife and tourism outcomes but with security concerns in conflict regions

Sean Denny, Gabriel Englander, Patrick Hunnicutt

Corresponding Authors: Sean Denny, Gabriel Englander
E-mail: smdenny@bren.ucsb.edu, aenglander@worldbank.org

This PDF file includes:

Supporting text
Figs. S1 to S18
Tables S1 to S7
SI References

Supporting Information Text

1. Supplementary Methods

Each subsection details the construction of an outcome variable or set of outcome variables, the regression(s) on that outcome variable presented in the main text, and robustness checks for that outcome variable.

A. Elephant Poaching (Figure S1 and Table S6). The Monitoring the Illegal Killing of Elephants (MIKE) program records elephant deaths each year at sites across Africa and Asia. As of January 2023 when we downloaded the data, there were 96 sites, with data spanning from 2000 to 2021 (1). Among the 66 African MIKE sites, 8 are currently managed by AP (treatment group), and 35 were among those identified by AP as "anchor areas" meeting their criteria for future management (control group). Our regression analysis includes 578 site-year observations from these 43 distinct sites (Figure S1). Table S6 displays Average Treatment effects on the Treated (ATTs) by "cohort", or the calendar year in which protected areas are transferred to AP management

MIKE data contain the total number of elephant carcasses discovered at a site in a year, as well the number of those carcasses that were killed illegally (poached). Dividing the number of poached carcasses by the total number of carcasses yields the Proportion of Illegally Killed Elephants (PIKE). This normalization controls for fluctuating survey efforts and elephant population sizes across sites and years under the following assumption: conditional on the number of poached and non-poached carcasses available for discovery, the probability of finding a poached carcass equals the probability of finding a non-poached one (2, 3). Studies of MIKE sites corroborate that PIKE accurately reflects mortality patterns in the sense that PIKE is not confounded by changes in survey effort or in the underlying elephant population level (4, 5).

Even if the conditional probabilities of finding poached and non-poached carcasses differ, bias in our estimated impact of AP management on PIKE would only arise if AP management changes the probability of discovering a poached carcass relative to a non-poached one. AP management could theoretically increase or decrease the relative probability of finding a poached carcass. When a protected area has few rangers, all patrol effort may occur in "hotspot" locations where finding poached carcasses is likeliest. If AP increases the number of rangers on staff compared to government-managed protected areas, those additional rangers could patrol locations where finding poached carcasses is less likely, and finding non-poached carcasses more likely. This change in where rangers patrol would mechanically reduce PIKE, even if the protected area's true rate of poaching hasn't changed. On the other hand, AP could increase the relative probability of finding poached carcasses if it is better able to target ranger patrols to hotspot locations. This change in where rangers patrol would mechanically increase PIKE. While we cannot test for changes in where rangers patrol due to these data being non-public, we can test whether AP management affects the probability of reporting any MIKE data in a given year. We find no evidence that AP management significantly influences the probability of reporting carcass data to MIKE: the ATT is -0.055 with a standard error of 0.142.

Figure S1 exhibits an increasing trend in elephant poaching in the years prior to transference. In the absence of transference to AP management, elephant poaching may have continued to increase. In this scenario, our ATT of -0.153 would underestimate the true reduction in elephant poaching due to AP management. We use the R package HonestDiD to implement the approach described in Section 2.4.3 of Ref. (6) in order to quantitatively assess this bias. Under the scenario that the pre-trends would have continued in the absence of transference to AP management, this approach considers how the average trend in the pre-period coefficients would extrapolate across the post-period and change the treatment coefficients and confidence intervals. The resulting confidence interval accounts for both estimation error in the treatment coefficients and in the pre-trend. The 95% confidence interval is (-0.728, -0.286), indicative of a larger reduction in elephant poaching than our ATT. Thus, the pre-trends in Figure S1 do not invalidate our inference that AP management reduces elephant poaching.

A.1. Spillovers. A separate concern relates to spillovers to control protected areas. Since AP seems to increase monitoring and enforcement, PIKE could increase in control protected areas if elephant poachers reallocate their effort away from protected areas managed by AP. This increase in poaching in control areas because of AP management would cause us to overestimate the reduction in poaching that is due to AP. We test for this possibility by identifying "spillover" protected areas.

For each area transferred to AP, we identify the geographically nearest control area, and we assign the control area the same transference year as the protected area that was actually transferred to AP. We repeat our primary specification with these spillover protected areas as the treatment group, excluding the areas managed by AP from the regression. The control group in this regression therefore contains areas that are less likely to be affected by AP's activities because they are geographically farther from areas managed by AP. The spillovers appear to operate in the opposite direction from that described above. Instead of increasing following transference of a nearby area to AP management, PIKE decreases in nearby spillover areas, though the coefficient is not statistically significant (ATT = -0.099 with a standard error of 0.096). It is therefore unlikely that spillovers cause us to overestimate the reduction in PIKE due to AP management; if anything, we may be underestimating the reduction.

As an additional assessment of the influence of spillovers on our main result in Table S3, we repeat our primary specification excluding spillover protected areas from the control group. The treatment group is areas transferred to AP management, and the control group is areas geographically farther from AP-managed areas. We obtain a similar estimate as our main result (ATT = -0.146 with a standard error of 0.082, compared to our main result of ATT = -0.153 with a standard error of 0.069).

B. Bird Abundances (Figures S2 to S7 and Tables S4 and S7). eBird, a citizen science platform for birding enthusiasts, captures species-specific observations from birding trips across the globe (7). During each birding trip, observers record the quantity

of bird species they encounter. Further details, such as the number of observers participating in the trip, the hours spent birding, and a geotagged timestamp, enrich the dataset. This geolocation data enables us to identify birding trips within specific protected areas, and the trip duration and number of observers data allow us to control for survey effort.

Contrary to our other analyses, our unit of observation in this case is the individual birding trip. This choice is informed by our adoption of the main specification from a recent study that examined the relationship between air pollution regulation and bird abundances using eBird data (8). Replicating this established specification limits our researcher degrees of freedom. In this specification, the dependent variable is the log total number of birds observed on a trip, and the control variables are duration of the birding trip in hours, the number of observers in the birding party, and fixed effects for hour of day, protected area, and calendar year. We also follow Ref. (8) in dropping the top 1% of bird abundance observations in order to remove outliers. We imputed missing values of birding trip duration and number of observers using their respective means calculated from non-missing values within our dataset, which encompasses eBird observations inside our protected areas of interest. We add as control variables three indicators for whether birding trip duration, number of observers, or hour of day were missing, which allows us to retain these observations in our regression. This regression employs 145,200 observations drawn from 20 treatment group protected areas and 106 control group areas between 1998 and 2022. Despite differences in the unit of observation and control variables, this regression applies the same dynamic difference-in-differences estimator as in our other regressions (9). Table S7 displays cohort-specific ATTs. Four cohorts have negative ATTs; however, these cohorts contribute only 11% of treatment observations.

Figure S2 exhibits a decreasing trend in bird abundances in the years prior to transference. In the absence of transference to AP management, bird abundances may have continued to decrease. In this scenario, our ATT of -0.318 would underestimate the true increase in bird abundances due to AP management. We use the R package HonestDiD to implement the approach described in Section 2.4.3 of Ref. (6) in order to quantitatively assess this bias. Under the scenario that the pre-trends would have continued in the absence of transference to AP management, the resulting 95% confidence interval is (1.350, 1.730), indicative of a larger increase in bird abundances than our ATT. Thus, the pre-trends in Figure S2 do not invalidate our inference that AP management increases bird abundances.

When we restrict the eBird data to complete checklists only, the number of observations falls to 92,574. We obtain similar results in this case (ATT = 0.306 with a standard error of 0.075, compared to our main specification ATT of 0.318 with a standard error of 0.072).

B.1. Robustness to Changes in Birder Locations (Figure S3). We consider three threats to our interpretation that the post-transference increase in bird abundance per trip represents a true increase in the population of birds because of AP management. First, if AP shifts where birding trips occur within its protected areas toward locations with greater bird abundance, we would overestimate the increase in bird abundances due to AP management. In this case, even if the true bird population of a protected area did not change, we would estimate an increase in abundance because of the shift in the composition of where birder observations are occurring. One reason this shift might occur is if AP tourism operations are more skilled at bringing visitors to locations with abundant wildlife, compared to if that protected area had continued to be managed by a government.

To assess this possibility, we identify locations within each protected area with above median bird abundances. We do so by regressing log bird abundance per trip on trip duration, number of observers, hour of day fixed effects, indicators for whether each of trip duration, number of observers, and hour of day are missing, calendar year fixed effects, and protected area fixed effects. We save the residuals from this regression. Then we calculate the average value of the residuals over all time periods for each 0.1° grid cell in each protected area. These residuals represent bird abundances net of birder effort and hour of day, calendar year, and protected area constants. Then we identify the grid cells whose average residual is above the protected area's median grid cell value. If we detect a greater proportion of birder observations occurring in these grid cells post-transference, that would indicate that AP is changing birders' locations in a way that would cause us to overestimate the effect of AP management on bird abundances.

We implement our dynamic difference-in-differences estimator at the level of 0.1° grid cell-protected area-year. We cluster standard errors at the level of protected area, and the only control variables are calendar year and grid cell-protected area fixed effects. The dependent variable is an indicator that equals 1 if two conditions are met and equals 0 if either condition is not met. The two conditions are (1) the grid cell has above median bird abundance and (2) the proportion of birder observations in that grid cell-year is above the protected area's median proportion (median calculated across all the protected area's grid cells and years).

Prior to transference, there is no trend in the probability that the proportion of observations in bird-abundant grid cells is above the median (Figure S3a). However, in the first two years of AP management, there is a statistically significant increase, and this effect persists on average over the eleven years of AP management we consider (ATT = 0.029 with a standard error of 0.012). It appears that part of the post-transference increase in bird abundances is due to a greater share of observations occurring in more bird-abundant places within protected areas.

How large is this upward bias? Since AP increases by 2.9 percentage points the share of observations in bird-abundant cells, we replace this share of observations with values from cells with below median bird abundance and then re-estimate our primary specification. Recall that the unit of observation in our primary specification is a birder trip. This regression uses data that have been corrected for the compositional change that occurred post-transference. For example, suppose 50% of an area's post-transference observations occur in grid cells with above median bird abundances. For 5.8% of these observations, we randomly replace the log bird abundance and effort control variables with the mean values of the protected area's below median abundance grid cells. The resulting share of post-transference observations "in" bird-abundant grid cells is now 2.9

percentage points lower because 5.8% $\times$ 50% = 2.9%.

Our main result—AP management increases bird abundances—is robust to using corrected data that holds constant the share of observations in bird-abundant locations post-transference. The regression coefficients are quite similar to those from our primary specification (Figure S3b). The average effect remains large and statistically significant (ATT = 0.277 with a standard error of .072), which represents an increase in bird abundances of 32% (compared to 37% in our primary specification).

B.2. Robustness to Changes in Birder Seasonality (Figure S4). The second concern parallels the first, except rather than studying whether AP shifts *where* birding trips occur we study whether AP shifts *when* birding trips occur. Bird abundances naturally fluctuate over the course of a year due to seasonal bird migration. If AP increases the share of birding trips that occur in bird-abundant months of the year, then we would overestimate the increase in bird abundances due to AP management. To assess this possibility and its implications for our main result, we implement the identical procedure described in SI B.1, except now month of year is the dimension of interest rather than a 0.1° grid cell. Month of year refers to the same month every year (e.g., "January", as opposed to "January 2018").

Prior to transference, there is little trend in the probability that the proportion of observations in bird-abundant months of the year is above the median (Figure S4a). However, there is a statistically significant increase on average over the eleven years of AP management we consider (ATT = 0.046 with a standard error of 0.015). It appears that part of the post-transference increase in bird abundances is due to a greater share of observations occurring in protected areas' more bird-abundant months of the year.

We assess the magnitude of this upward bias in the same manner as in SI B.1. Since AP increases by 4.6 percentage points the share of observations in bird-abundant months of the year, we replace this share of observations with values from months of the year with below median bird abundance and then re-estimate our primary specification. For each protected area transferred to AP management, we randomly replace the log bird abundance and effort control variables with the mean values of the protected area's below median abundance months of the year until the resulting share of post-transference observations "in" bird-abundant months of the year is 4.6 percentage points lower.

Our main result is robust to using corrected data that holds constant the share of observations in bird-abundant months of the year post-transference (Figure S4b). The average effect remains large and statistically significant (ATT = 0.240 with a standard error of 0.072), which represents an increase in bird abundances of 27%. Correcting for the compositional change in seasonality decreases the ATT by 0.078 log points (from 0.318 to 0.240). Recall from SI B.1 that correcting for the compositional change in location decreased the ATT by 0.041 log points (from 0.318 to 0.277). If the effects of the two compositional shifts were additive, we would obtain an ATT of 0.199, representing an increase in bird abundances of 22%.

B.3. Robustness to Changes in Birder Skill (Figure S5). The third concern relates to changes in who visits protected areas post-transference. If eBird observations are more likely to be submitted by individuals who are more skilled at observing birds or are more likely to report having seen a greater number of birds, then the post-transference increase in bird abundance could be due to changes in birder composition rather than representing an increase in the population of birds.

Our primary dataset of bird abundance within treatment and control protected areas includes observations submitted by 8,255 unique birders. We download all eBird data for Africa between 1998 and 2022. We filter the data to observations submitted by our 8,255 birders outside our treatment and control protected areas. That is, we define birder skill using different data than that in our primary specification. We shape these data with the same steps we used to create our primary dataset (dropping the top 1% of bird abundance observations, imputing missing effort variables with mean values, and creating indicator variables for initially missing values). We regress log bird abundance on trips outside our treatment and control protected areas on trip duration, number of observers, hour of day fixed effects, indicators for whether each of trip duration, number of observers, and hour of day are missing, and calendar year fixed effects. We save the residuals and calculate the average value of the residuals for each birder. A birder's average residual value represents their skill (or alternatively, the excess number of birds they typically report) because the residuals are the log number of birds reported net of birder effort and hour of day and calendar year constants. Finally, we calculate the median residual value across all birders.

We then create a new variable in our primary dataset: an indicator that equals 1 if the observation is from an above median skill birder and equals 0 otherwise. We assume the 700 birders with zero observations outside our treatment and control protected areas are below median skill. We repeat our dynamic difference-in-differences estimator with this indicator as the dependent variable. Unlike in our primary specification, the only control variables are calendar year and protected area fixed effects. Rather than estimating an increase in birder skill, which would indicate that the post-transference increase in bird abundance is an artefact of a change in the composition of birders, we find a statistically significant decrease in the probability that an eBird observation is submitted by an above median skill birder (Figure S5). While some regression coefficients are positive, others are negative, and the ATT of -0.277 (with a standard error of 0.024) is large in magnitude compared to the mean of the dependent variable in control areas of 0.446. AP may make their protected areas more accessible to less experienced birders, which would accord with our findings that AP increases tourism. Unlike the robustness checks in SI B.1 and B.2, accounting for the compositional change in birders would increase our estimate of the effect of AP management on bird abundances.

B.4. Flexibly Controlling for Birder Effort (Figure S6). In addition to our primary analysis, we conducted a robustness check to control for survey effort more flexibly, again replicating a previously implemented procedure to limit our researcher degrees of freedom (8). This procedure begins by creating linear, squared, and cubed terms for duration of birding trip in hours, numbers of observers, distance covered in km, and area covered in hectares, then interacting these variables with each other while still

retaining the non-interacted individual variables as potential predictors. Furthermore, we constructed dummy variables to account for cases where the distance covered was 0 km (representing a stationary count) and instances where only one observer was present.

Following this, we utilized a Least Absolute Shrinkage and Selection Operator (LASSO) with 10-fold cross-validation, resulting in 5 retained survey effort control variables with non-zero coefficients at the optimal shrinkage penalty. The dependent variable in the LASSO regression is log bird abundance per trip, and to the survey effort control variables mentioned as being created above we also included years to transference to AP in event time and fixed effects for hour of day, protected area, and calendar year, since these variables will be included in the subsequent dynamic difference-in-differences regression to estimate the effect of AP management. We imputed missing values of predictor variables with their respective means.

Finally, we re-estimated our dynamic difference-in-differences model, this time controlling for the 5 survey effort variables identified by LASSO, as well as fixed effects for hour of day, protected area, and calendar year (Figure S6). The ATT of AP management on log bird abundance in this specification is 0.120 (standard error = 0.078). While still representing a large magnitude increase in bird abundances, this average effect is smaller than in our preferred specification because the five years after transference coefficient is more negative. However, 9 out of the 10 other post-transference coefficients are large and positive, and similar in sign and magnitude to our preferred specification in Figure S2.

B.5. Effect of AP Management on Number of Bird Species (Figure S7 and Table S4). We replicate our primary specification with log(number of unique bird species observed) per trip as the dependent variable, instead of log(number of birds observed). Other than the different dependent variable, the data and regression specification in Figure S7 is identical to that of Figure S2. The regression coefficients in Figure S7 are quite similar to those in Figure S2. There is a downward trend in the pre-period coefficients, suggesting that biodiversity is decreasing in the protected areas that will be transferred to AP. Post-transference, there is an immediate increase in the number of bird species observed, which persists for most years of AP management. The main exception to this persistent increase in bird species is the coefficient representing the effect five years after the beginning of AP management. This coefficient is also large and negative in Figure S2, but in the case of Figure S7 it may be offsetting the other positive post-transference coefficients and resulting in a slightly negative ATT (-0.107 with a standard error of 0.053). When we consider separate ATTs by the calendar year in which protected areas are transferred to AP, which are called "cohorts", we find that the four cohorts with the highest number of observations—comprising more than 70% of treatment group observations—all demonstrate positive ATTs, as shown in Table S4.

C. iNaturalist Tourist Visits (Figures S8, S9, and S11a). iNaturalist, akin to eBird, functions as a citizen science platform where both amateurs and researchers document their wildlife encounters (10). However, iNaturalist expands upon eBird's focus on avian life by including observations of all flora and fauna. Notably, unlike eBird, iNaturalist does not contain survey effort data, which renders it unsuitable as a source of wildlife data for our study. Nonetheless, the geolocated and timestamped observations in the iNaturalist database enable us to use it as a proxy for visits to protected areas.

Given that iNaturalist does not represent a comprehensive record of tourist visits, we configure the dependent variable as an indicator that equals 1 if any iNaturalist observations occur within a protected area in a specific year, and 0 otherwise. Consequently, the dependent variable in this analysis offers an extensive margin measure of whether any iNaturalist user visits took place.

There are no missing values in the data underlying the regression visualized in Figure S8. If a protected area receives no iNaturalist visits in a given year, the dependent variable simply registers as 0. The data we construct span the years 1998 to 2022. There are 3,625 observations because we have 145 protected areas (22 treatment group and 123 control group areas). The dependent variable's mean value among control areas is 0.462, which means that in 46% of area-years, at least one iNaturalist observation was recorded within the boundaries of the protected area during that year.

While the majority of iNaturalist data is likely recorded by tourists, it is important to note that protected area staff can also upload wildlife observations to iNaturalist. If AP staff are more likely to upload observations than their counterparts at other protected areas, this would upwardly bias our estimate of the effect of AP management on tourist visits. To test the robustness of our results to the potential inclusion of protected area staff in iNaturalist data, we implement the following approach. We exclude all data uploaded by any iNaturalist user who records an observation inside the same protected area between 30 and 365 days from their last visit, as such users could plausibly be protected area staff. Reconstructing the panel data as per our primary specification results in the same 3,625 observations, but reduces the dependent variable's mean value among control areas to 0.352. Nonetheless, our analysis yields a similar result to our primary specification, indicating that potential inclusion of protected area staff data does not cause bias. The pre-trend remains flat and the ATT is 0.175, with a standard error of 0.040 (Figure S9).

We also return to our primary iNaturalist data and repeat our regression with log(number of iNaturalist visits per year) as the dependent variable (Figure S11a). Because the dependent variable is a natural logarithm, we restrict the data to area-years with positive visits. The number of observations in the regression is therefore 1,635. We obtain an ATT of 0.060 with a standard error of 0.037.

D. eBird Tourist Visits (Figures S10 and S11b). Figure S10 employs a similar analytical approach to Figure S8, now using eBird visits as an indicator of tourism. The dependent variable again takes the value of 1 if any eBird observations are recorded within a protected area in a particular year, and 0 otherwise.

Unlike the previous bird abundance analysis (Figure S2), which leverages eBird data at the level of individual birding trips, this analysis utilizes data at the level of protected area-year. This approach enables us to understand eBird data in terms of visits to specific protected areas over time.

There are 3,625 observations in the regression because the data span the years 1998 to 2022 and there are 145 protected areas. The mean value of the dependent variable in control protected areas is 0.510.

We also repeat our regression with log(number of eBird visits per year) as the dependent variable (Figure S11b). Because the dependent variable is a natural logarithm, we restrict the data to area-years with positive visits. The number of observations in the regression is therefore 1,767. We obtain an ATT of 0.259 with a standard error of 0.096.

E. Economic Development (Figure S12). We measure economic development using data on "asset wealth". Atlas AI provided asset wealth data to us at an annual frequency and delineated by second-level administrative divisions, spanning the years from 2003 to 2021 (11, 12). We filter the data to include only those administrative divisions that are located within a 25 km radius of our treatment and control protected areas. When multiple administrative divisions intersect a protected area, we weight asset wealth across administrative divisions by their area of overlap with the protected area. The resulting panel data set consists of 2,755 observations, because we observe 145 protected areas over 19 years. Given that the asset wealth index is unitless, we standardize asset wealth across all protected area-years. This involves subtracting the mean asset wealth and then dividing by the standard deviation of asset wealth. Figure S12a illustrates the mean standardized asset wealth for each protected area included in our data set, and Figure S12b displays the dynamic difference-in-differences estimates.

F. Conflict (Figures S13 to S18). The Armed Conflict Location and Event Database Project (ACLED) uses reports from local, national, and international sources to generate geocoded event data on conflict around the world. Data from ACLED document numerous features of conflict events, including their timing, location, type, and the involved actors. ACLED prioritizes external validity in its data collection protocol and therefore captures a wider range of conflict types in comparison to other sources of conflict event data (13).

As of May 30, 2023, ACLED has documented over 306,000 conflict events on the African continent alone. This population of events forms the basis of the outcome measures we use to estimate the effect of AP management on conflict. We exclude from these data conflict events that cannot be geolocated to the town-level to minimize measurement error when determining where conflict occurred relative to protected areas' boundaries and corresponding buffer areas. We also exclude from the ACLED data conflict events classified as strategic developments ("contextually important events which may contribute to a state's political disorder and/or may trigger future events"). Such events are contextually-defined and may not involve actual violence. With these inclusion criteria, our final sample of ACLED data include 214,534 unique conflict events in Africa between 1998 and 2022. 45% of these events are protests and riots, 24% are battles, and 23% involve civilian targeting (violence against civilians), approximately.

We rely on ACLED's interaction codes to measure the presence and extent of different forms of civilian targeting in and around protected areas in our sample. ACLED events with interaction codes ending in 7 designate violence against civilians and thus form the basis of our civilian targeting outcome. ACLED events with an interaction code of 27 designate rebel-led attacks on civilians and thus form the basis of our rebel-perpetrated civilian targeting outcome. ACLED events with an interaction code of 17 designate government-led attacks on civilians and thus form the basis of our government-perpetrated civilian targeting outcome. ACLED events with an interaction code of 37 or 47 designate militia-led attacks on civilians and thus form the basis of our militia-perpetrated civilian targeting outcome. ACLED recorded 49,287 instances of civilian targeting in Africa between 1998 and 2022. 23% of these events were perpetrated by rebel groups, 23% were perpetrated by government forces, and 64% were perpetrated by militias, approximately.

Our temporal unit of observation is the year, and our spatial unit of observation is the area within a protected area's boundaries plus the area contained within a 25-kilometer buffer of a protected area's boundaries. Figure S13 displays results for each of our three conflict measures.

As with our tourism analysis, we primarily use binary measures of conflict in order to reduce measurement error stemming from reporting bias. Imagine there is no difference in the annual level of conflict experienced between AP and government-managed protected areas, yet we estimate a positive effect because AP makes it easier for media sources to detect and report on conflict events. This possibility suggests the different annual levels of conflict we observe between AP and government-managed areas may be inaccurate. By comparison, differences in the presence of conflict between the two groups should be less susceptible to reporting bias, so long as AP does not change the probability of media outlets detecting and reporting on any conflict at all. We support this assumption by documenting that AP management does not affect the presence of any form of conflict on average (Figure S15). Focusing on the presence (or onset) of conflict also aligns with prior research investigating armed group behavior (14, 15).

F.1. Understanding the Increase in the Presence of Civilian Targeting (Figure S17). Why might AP management increase the probability of violence against civilians (Table S3 and Figure S13a)? We propose that AP management restricts the ability of armed groups to generate revenue via natural resource extraction, subsequently displacing armed groups' revenue generation activities towards the civilian population (e.g., kidnapping and extortion). If AP's increased capacity for monitoring and enforcement is displacing armed groups' revenue generation activities, then some of the conflict events we observe in and around AP's protected areas

should involve armed groups known to exploit natural resources for revenue generation. Numerous qualitative descriptions of the conflict events occurring in and around protected areas after they are transferred to AP confirm this intuition:

- "Around 100 heavily armed poachers lead by LRA rebel killed a park ranger and 2 FARDC soldiers, and injured another ranger, patrolling the Garamba National Park during an ambush. 2 of the poachers were also killed, who were also allegedly made up of a number of foreigners, including a Sudanese army deserter. Reports conflicted as to the date of the attack." (ACLED Event ID: DRC9371)
- "An LRA rebel involved in poaching was killed by Garamba park guards." (ACLED Event ID: DRC10020)
- "On 26 July 2022, suspected JNIM militants and APN [African Parks Network] Park rangers exchanged gunfire in the village of Dassari (Materi, Atacora). There were no casualties." (ACLED Event ID: BEN739)
- "On 4 April 2022, overnight presumed (Jama'at Nusrat al Islam wal Muslimeen) JNIM or ISWAP abducted a farmer and a motor taxi driver from the Fulani community in the village of Kangara [Kangara Peulh], in Arrondissement of Birni-Lafia (Karimama, Alibori). The gunmen took the abductees to the interior of Park W." (ACLED Event ID: BEN655)
- "On 9 June 2021, presumed JNIM militants detained (some were tied up) and interrogated road users in the Arly National Park (Logobou, Tapoa)." (ACLED Event ID: BFO4773)
- "On 15 October 2022, unspecified security forces (described as 'soldiers', and provisionally coded as SSPDF) shot and wounded two people (reported to be from the Aliab Dinka community) and demolished 34 dwellings in Gumbo (Juba county, Central Equatoria state). An opposition politician from Awerial county has alleged that the security forces also stole money from the houses that were destroyed." (ACLED Event ID: SSD8852)
- "On 2 October 2022, three FARDC soldiers shot at a young businessman at his home in Kamanyola (Walungu, Walungu, Sud-Kivu), presumably to rob him. The man was wounded, but survived." (ACLED Event ID: DRC27036)

It is plausible that AP's law enforcement components are related to these conflict events. For example, rangers deployed in Garamba National Park could have increased their patrolling efforts once the park was transferred to AP, given the substantial resources AP dedicates to anti-poaching efforts (Table 1). Subsequently, AP rangers may have been more likely to discover armed groups engaged in poaching. The roadblocks, kidnapping, and extortion described above are also consistent with our proposed mechanism, lending further credence to our finding of an increase in the probability of civilian targeting. Both government forces and rebel groups active in the regions where AP manages protected areas use these strategies to generate revenue, especially when exclusively controlling areas rich in natural resources is difficult (16, 17). Moreover, JNIM—the rebel group responsible for some of the civilian targeting described above—is known to rely on the trafficking of natural resources to generate revenue (18, 19).

Another observable implication of the displacement mechanism we propose is a post-transference increase in the probability of battles between rebel and government forces. These armed groups frequently compete for control over resource-rich regions in some of the countries where AP manages protected areas (20, 21). AP may exacerbate this competition in at least two ways. First, AP's increased monitoring and enforcement may result in more confrontations between rebel groups and AP rangers. ACLED codes AP rangers as government forces. We provide qualitative evidence of this above (see ACLED Event ID DRC9371 and BEN739). Second, AP's increased monitoring and enforcement may reduce the amount of resources within protected areas that government and rebel forces can extract without detection. Fighting between rebel and government forces may subsequently increase, as both groups seek control over the resources in protected areas that they can extract without triggering a response from AP's anti-poaching units. By comparison, battles involving local militias—who often vie for control over shared natural resources that are vital for subsistence but hold little monetary value as trafficked goods (e.g., water, arable land) (22)—should not become more likely post-transference, if the displacement mechanism we propose is operative.

Indeed, we find evidence that AP management increases the probability of battlefield confrontations between government and rebel forces (Figure S17a). Government and AP managed protected areas appear equally likely to experience battles between government and rebel forces in the pre-transference period. Once protected areas are transferred to AP, the probability of government-rebel battles increases by 6.5 percentage points on average (this ATT's standard error is 2.2 percentage points). In contrast, transferring protected areas to AP appears to have no average effect on the probability of battles between local militias and government forces (Figure S17b) and local militias and rebel forces (Figure S17c).

F.2. Selecting Buffer Sizes (Figure S18). There is no standardized spatial unit of observation in conflict research. Some researchers rely on political boundaries like administrative units to define their unit of observation (23), while others adopt a gridded data structure (24) that can vary in size (25).

Given this ambiguity, we provide here some conceptual justification for defining our unit of observation as the area within a protected area's boundaries plus a buffer area surrounding it, where the radius of the buffer area is 25 kilometers. The plausible mechanisms through which AP might affect conflict are quite local in scale. For example, we argue above that AP plausibly increases the probability of civilian targeting because it reduces the trafficking of natural resources armed groups relied on for revenue generation pre-transference, making armed groups more likely to loot civilians post-transference. Extant research suggests this looting takes place in the same location where armed groups previously engaged in natural resource extraction (23), rather than in distant locations where armed groups could have relocated to engage in revenue generation. Consistent

with this assertion is armed groups' tendencies to establish roadblocks near deposits of valuable natural resources that they do not fully control (16). Moreover, the decentralized nature of armed groups in the areas where AP operates (26) implies AP is more likely to shape local conflict dynamics than regional conflict dynamics. Absent a high degree of centralization, it seems implausible that one contingent of an armed group operating near an AP site could recuperate lost revenue resulting from AP's activities by coordinating with another contingent to increase the looting of civilians elsewhere.

We test how the relationship between AP and conflict changes when we alter the buffer area used to define our unit of observation (Figure S18). Removing the buffer area altogether largely produces similar results to our main analysis: transference to AP is not significantly related to the probability of protests and riots, increases the probability of civilian targeting (now, at the ten-percent level), and increases the probability of battles (now, at the five-percent level). For the probability of battles and the probability of protests and riots, increasing the buffer radius to 50 kilometers or 75 kilometers produces similar results to our main analysis. However, the effect of transference to AP on the probability of civilian targeting becomes statistically indistinguishable from zero when we increase the buffer radius to 50 kilometers, and then becomes negatively signed and statistically significant when we increase the buffer radius to 75 kilometers. For the reasons described above, we caution against attributing the 75 kilometer decrease in civilian targeting to AP management. The downward shift in these estimates may reflect how larger buffers include in our analysis urban areas where violence against civilians in some African countries is more likely, all else equal (27).

G. Mechanisms and Management Effectiveness Tracking Tool (METT) data (Table 1). METT data contain 3,999 self-assessments of management effectiveness for 2,577 protected areas between 1999 and 2016 (28). Six protected areas currently managed by AP have METT data for multiple years (treatment group), as do 27 protected areas identified by AP as anchor areas meeting their criteria for future management (control group). The six treatment group protected areas with multiple observations are Pendjari National Park (Benin), W National Park (Benin), Odzala-Kokoua National Park (Republic of Congo), Garamba National Park (Democratic Republic of the Congo), Bangweulu (Zambia), and Kafue National Park (Zambia). The data are so sparse that applying the dynamic difference-in-differences estimator we use for all other analyses results in an error. Consequently, we estimate a two-way fixed effects differences-in-differences regression instead (equivalently, a before-after-control-impact analysis). For a measure of management effectiveness Y in protected area i in calendar year t, we estimate the following equation with ordinary least squares regression:

$$Y_{it} = \beta AP_{it} + \gamma_i + \delta_t + \alpha X_{it} + \epsilon_{it} \quad [1]$$

where AP_{it} equals 1 if protected area i was managed by AP on or after year t, γ_i are protected area fixed effects, δ_t are year fixed effects, X_{it} is a matrix of other control variables, and ϵ_{it} is the error term. The protected area fixed effects control for all time-invariant characteristics of each protected area, such as physical geography, while the calendar year fixed effects account for time-varying factors that affect all protected areas uniformly, such as international conservation priorities and funding availability. As in all other analyses, we cluster standard errors at the level of protected area. The coefficient of interest is β, which captures the change in Y due to transference to AP management.

The matrix X_{it} first includes an indicator for whether the protected area receives funding from the Global Environment Facility (GEF), which can influence scores if respondents believe future GEF funding depends on the scores they report. The matrix also includes indicators for who participated in responding to the questionnaire, as these identities can also influence scores reported. For example, participation from local community members could result in lower scores on average if these individuals tend to be less satisfied with protected area management than protected area managers themselves. Specifically, X_{it} includes dummy variables for whether any of the following types of people were in the group that submitted the questionnaire: protected area managers, protected area staff, other protected area agency staff, NGO staff, members of the local community, donors, external experts, and other individuals. We also include indicators for whether any of these variables were missing, which allows us to retain these observations in our regressions.

The primary data from which we form the Y_{it} variables are responses to 30 questions (29, 30). Valid answers range from 0 to 3. For example, for the third question, "Law enforcement", respondents record 0 to represent "No effective capacity/resources", 1 to indicate "There are major deficiencies in staff capacity/resources", 2 for "The staff have acceptable capacity/resources", and 3 for "The staff have excellent capacity/resources". Consequently, we exclude responses with a value greater than 3 from our analyses. We adopt the categorization of other researchers in grouping the 30 questions into four dimensions: Design and Planning, Capacity and Resources, Monitoring and Enforcement Systems, and Decision-Making Inclusiveness (30, 31). We calculate the mean response to questions in each category, resulting in four variables (one for each category). We standardize the category scores so that the regression coefficients are interpretable in terms of standard deviations.

2. Supplementary Figures

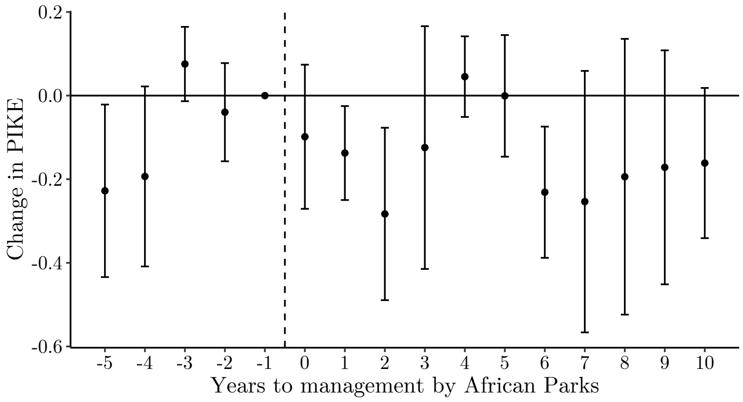

Fig. S1. AP management reduces elephant poaching. Points are regression coefficients, bars are 95% confidence intervals, and standard errors are clustered at the protected area level. The unit of observation is an area-year and the control variables are area fixed effects, year fixed effects, a third-order polynomial in precipitation in m, and 12 degree day bins (Materials and Methods). The dependent variable is the Proportion of Illegally Killed Elephants (PIKE) and the number of observations is 578 (SI A). The ATT corresponding to this figure is displayed in Table S3.

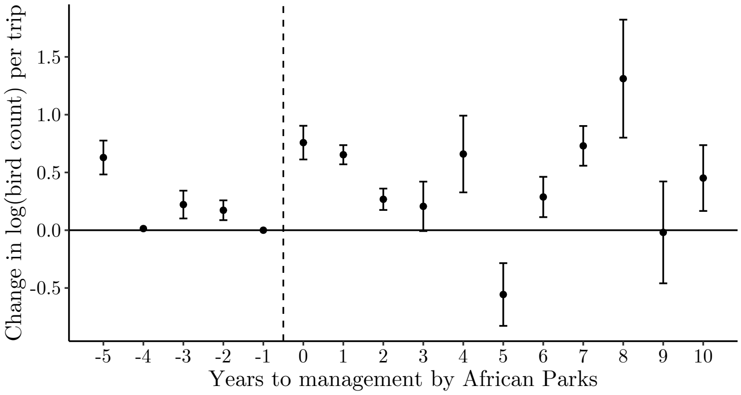

Fig. S2. AP management increases bird abundances. Points are regression coefficients, bars are 95% confidence intervals, and standard errors are clustered at the protected area level. The dependent variable is log(number of birds observed), the unit of observation is the birding trip, the number of observations is 145,200, and the control variables are trip duration in hours, number of observers in the birding party, and hour of day, area, and year fixed effects (SI B). The confidence interval for the four years before transference coefficient is omitted because it extends beyond the range of the figure. The ATT corresponding to this figure is displayed in Table S3

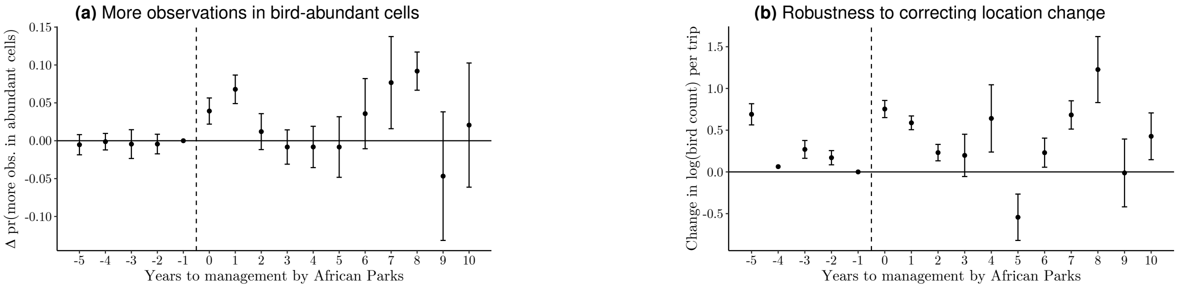

Fig. S3. AP management (a) increases the share of eBird observations occurring from bird-abundant cells, but (b) its positive effect on bird abundances remains after adjusting for this composition shift. Points are regression coefficients, bars are 95% confidence intervals, and standard errors are clustered at the protected area level. In (a), the unit of observation is the .1° grid cell-protected area-year, the number of observations is 51,400, and the only control variables are year and protected area-grid cell fixed effects. The dependent variable is an indicator that equals 1 if two conditions are met and equals 0 if either condition is not met. The two conditions are (1) the grid cell has above median bird abundance and (2) the proportion of birder observations in that grid cell-protected area-year is above the protected area's median proportion (SI B.1). In (b), the unit of observation is the birding trip, the dependent variable is log(number of birds observed), the number of observations is 145,200, and the control variables are trip duration in hours, number of observers in the birding party, and hour of day, area, and year fixed effects. The data are adjusted to hold constant the share of observations in bird-abundant locations post-transference (SI B.1). The ATT is 0.277 (standard error = .072). The confidence interval for the four years before transference coefficient is omitted because it extends beyond the range of the figure.

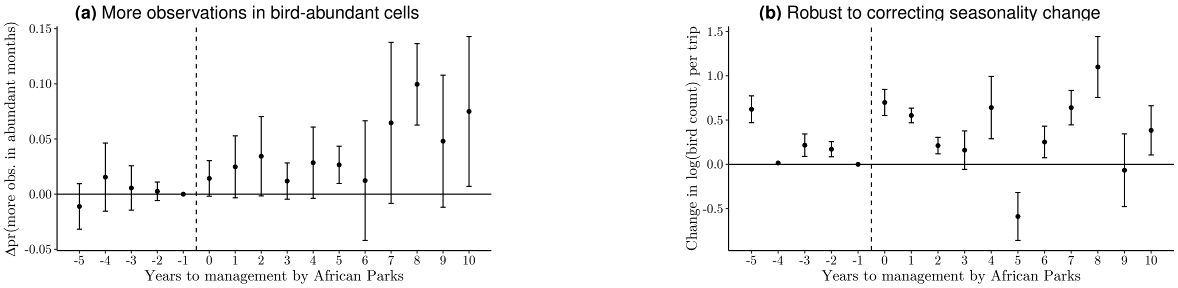

Fig. S4. AP management (a) increases the share of eBird observations occurring in bird-abundant months, but (b) its positive effect on bird abundances remains after adjusting for this compositional shift. Points are regression coefficients, bars are 95% confidence intervals, and standard errors are clustered at the protected area level. In (a), the unit of observation is the protected area-month-year, the number of observations is 37,800, and the only control variables are year and protected area-month of year fixed effects. The dependent variable is an indicator that equals 1 if two conditions are met and equals 0 if either condition is not met. The two conditions are (1) the month has above median bird abundance and (2) the proportion of birder observations in that protected area-month-year is above the protected area's median proportion (SI B.2). In (b), the unit of observation is the birding trip, the dependent variable is log(number of birds observed), the number of observations is 145,200, and the control variables are trip duration in hours, number of observers in the birding party, and hour of day, area, and year fixed effects. The data are adjusted to hold constant the share of observations in bird-abundant months post-transference (SI B.2). The ATT is 0.240 (standard error = .072). The confidence interval for the four years before transference coefficient is omitted because it extends beyond the range of the figure.

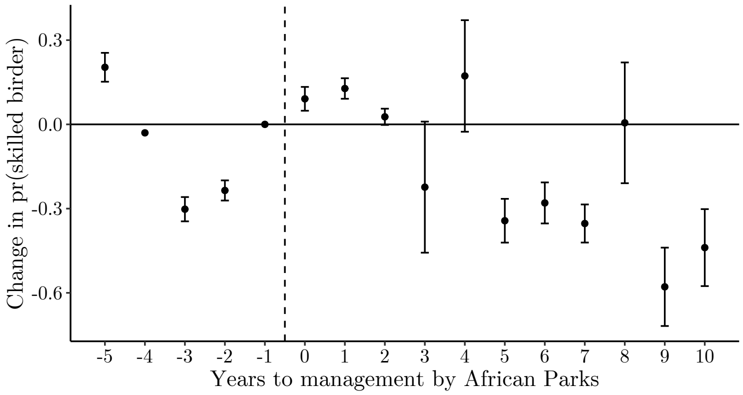

Fig. S5. Birder skill decreases following transference to AP management. Points are regression coefficients, bars are 95% confidence intervals, standard errors are clustered at the protected area level, the unit of observation is the birding trip, the number of observations is 145,202, and the control variables are area and year fixed effects. The dependent variable is an indicator that equals 1 if the observation is from an above median skill birder and equals 0 otherwise (SI B.3). The ATT is -0.277 (standard error $=$ 0.024). The confidence interval for the four years before transference coefficient is omitted because it extends beyond the range of the figure. The mean of the dependent variable in control areas of 0.446.

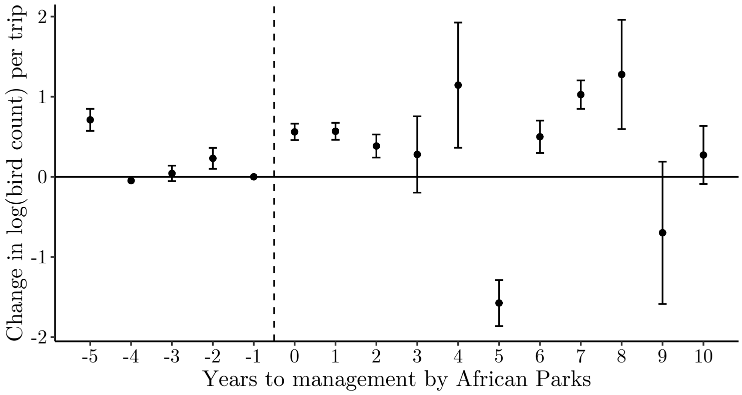

Fig. S6. Effect of AP management on bird abundance when choosing survey effort control variables with LASSO. This figure replicates the robustness check of Ref. (8), allowing LASSO to choose which survey effort variables to control for in the subsequent regression of log bird abundance on transference to AP management (SI B.4). The LASSO model primarily selected from interactions of linear, quadratic, and cubic functions of duration of birding trip in hours, number of observers, distance covered in km, and area covered in hectares. In total, LASSO retained 5 surveyor effort variables with non-zero coefficients; these variables were hence controlled for in the subsequent dynamic difference-in-differences regression, whose results are displayed here. We also control for hour of day, area, and year fixed effects in the dynamic difference-in-differences regression. Points are regression coefficients, bars are 95% confidence intervals, and standard errors are clustered at the protected area level. The unit of observation is the birding trip and the number of observations is 145,200. The confidence interval for the four years before transference coefficient is omitted because it extends beyond the range of the figure. The ATT is 0.120 (standard error = 0.078), which corresponds to an average increase in bird abundances of 13% due to AP management.

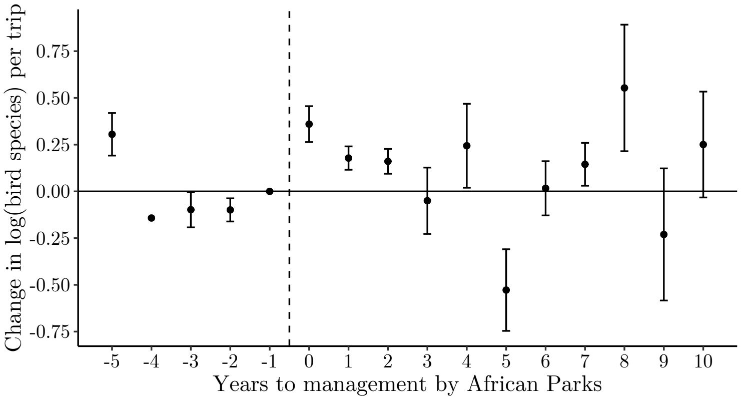

Fig. S7. Effect of AP management on number of bird species. This figure replicates the regression of Figure S2, except instead of log(number of birds observed) as the dependent variable, the dependent variable is log(number of unique bird species observed). Points are regression coefficients, bars are 95% confidence intervals, and standard errors are clustered at the protected area level. The unit of observation is the birding trip and the number of observations is 145,200. Section B.5 discusses this result further and Table S4 displays ATTs.

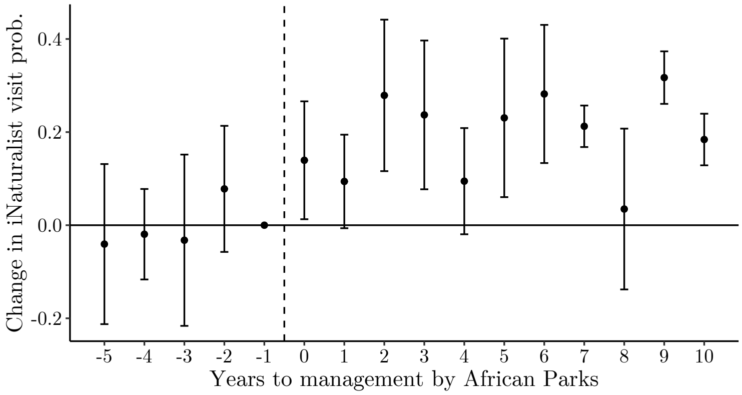

Fig. S8. AP management increases the probability of positive iNaturalist visits. Points are regression coefficients, bars are 95% confidence intervals, and standard errors are clustered at the protected area level. The unit of observation is an area-year and the control variables are area fixed effects, year fixed effects, a third-order polynomial in precipitation in m, and 12 degree day bins (Materials and Methods). The dependent variable is an indicator for positive iNaturalist observations inside a given protected-area year and the number of observations is 3,625 (SI C). The ATT corresponding to this figure is displayed in Table S3.

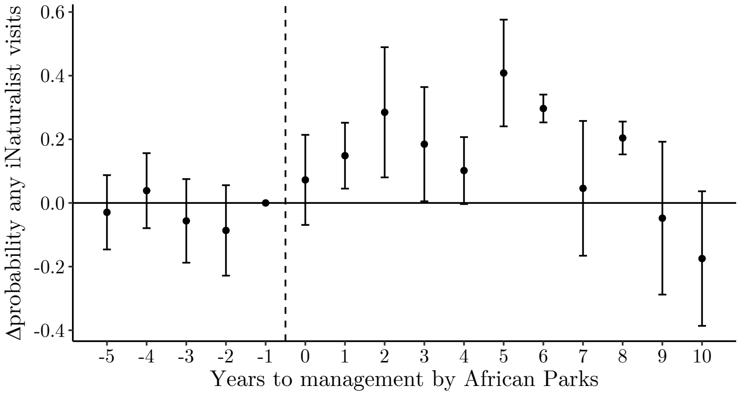

Fig. S9. Effect of AP management on probability of positive iNaturalist visits, excluding potential protected area staff. We exclude potential protected area staff from iNaturalist data, then repeat the procedure which produced Figure S8 (SI C). Points are regression coefficients, bars are 95% confidence intervals, standard errors are clustered at the protected area level, the unit of observation is the area-year, the number of observations is 3,625, and the control variables are area fixed effects, year fixed effects, a third-order polynomial in precipitation in m, and 12 degree day bins (Materials and Methods). The ATT is 0.175 (standard error = 0.040).

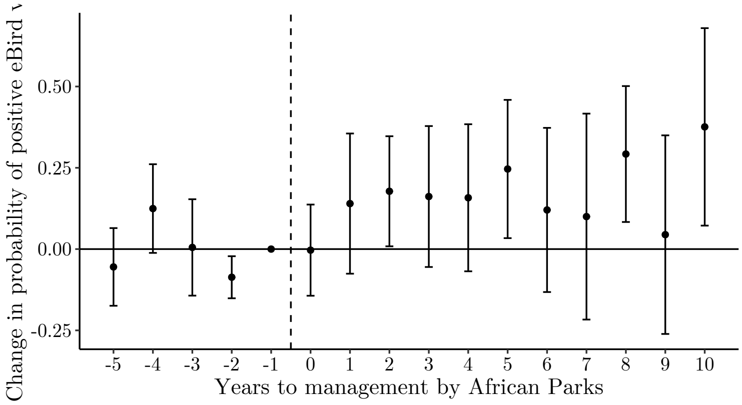

Fig. S10. AP management increases the probability of positive eBird visits. Points are regression coefficients, bars are 95% confidence intervals, and standard errors are clustered at the protected area level. The unit of observation is an area-year and the control variables are area fixed effects, year fixed effects, a third-order polynomial in precipitation in m, and 12 degree day bins (Materials and Methods). The dependent variable is an indicator for positive eBird observations inside a given protected-area year and the number of observations is 3,625 (SI D). The ATT corresponding to this figure is displayed in Table S3.

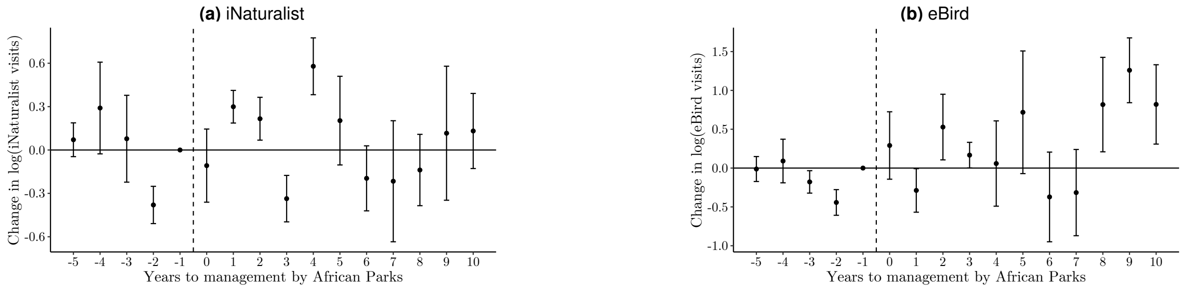

Fig. S11. Effect of AP management on (a) log(iNaturalist visits) and (b) log(eBird visits). Points are regression coefficients, bars are 95% confidence intervals, standard errors are clustered at the protected area level, the unit of observation is the protected area-year, and the control variables are area fixed effects, year fixed effects, a third-order polynomial in precipitation in m, and 12 degree day bins (Materials and Methods). Because the dependent variable in both regressions is a natural logarithm, data are restricted to area-years with positive visits. In (a), the number of observations is 1,635, the ATT is 0.060, and the standard error of the ATT is 0.037. In (b), the number of observations is 1,767, the ATT is 0.259, and the standard error of the ATT is 0.096.

(a) Mean asset wealth within 25 km of protected areas

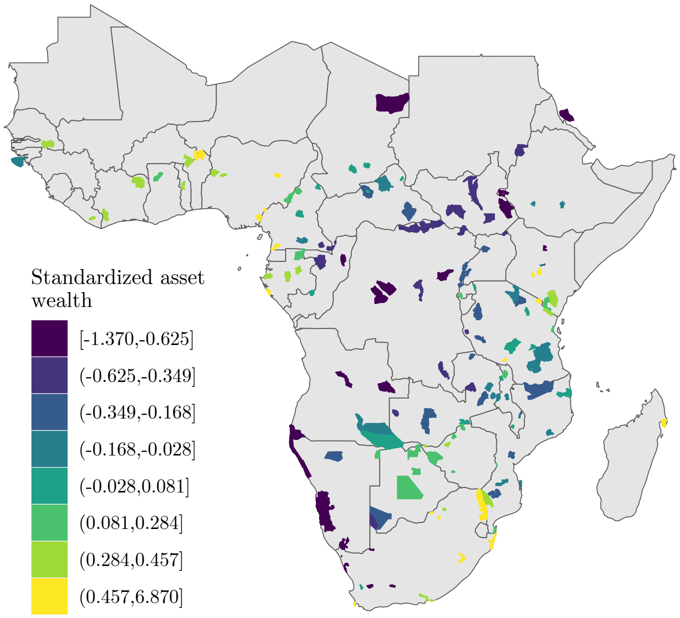

(b) Effect of AP management on asset wealth near protected areas

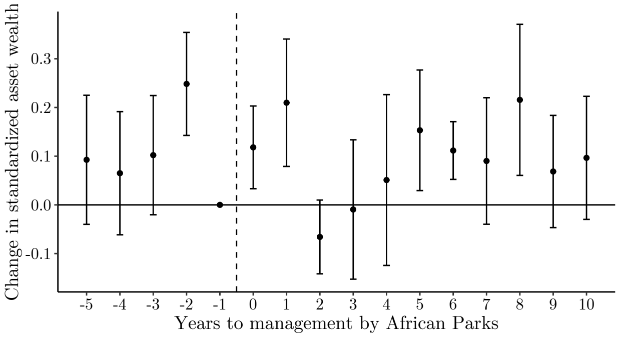

Fig. S12. Inconclusive effect of AP management on asset wealth. (a) This panel displays the mean standardized asset wealth within a 25 km radius of each protected area, as calculated from 2003 to 2021. (b) This panel illustrates the effect of AP management on asset wealth. The upward pre-trend prior to AP management means we cannot interpret the post-transference increase in asset wealth as being due to AP management. Points are regression coefficients, bars are 95% confidence intervals, and standard errors are clustered at the protected area level. The unit of observation is an area-year, the number of observations is 2,755, and the control variables are area fixed effects, year fixed effects, a third-order polynomial in precipitation in m, and 12 degree day bins (Materials and Methods). The ATT corresponding to this figure is displayed in Table S3.

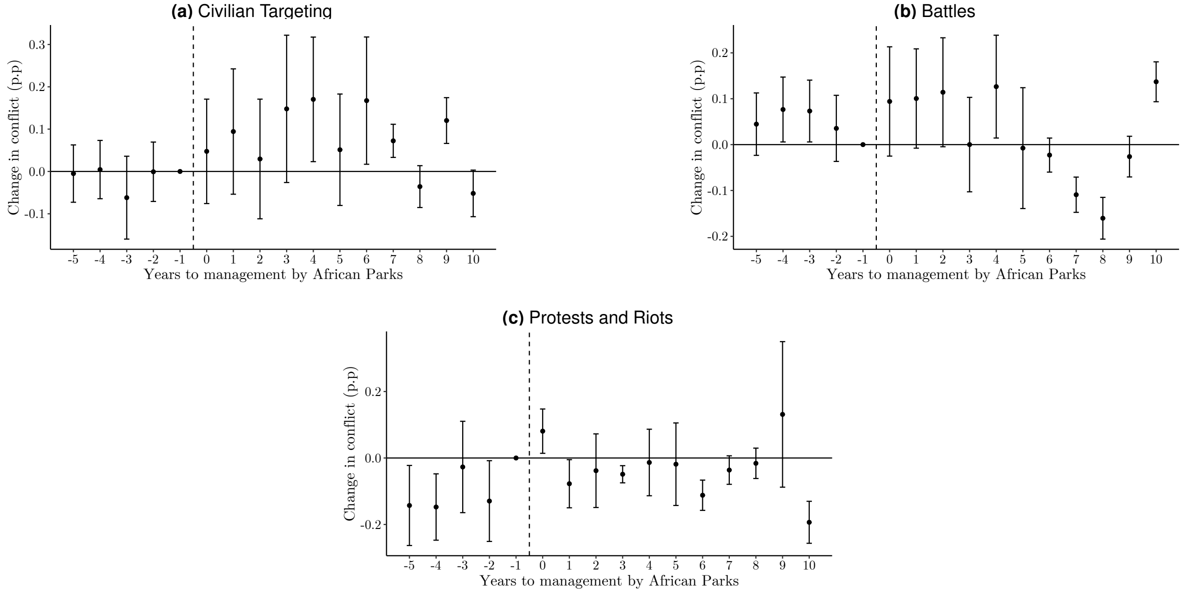

Fig. S13. AP management increases the (a) presence of civilian targeting, but not the (b) presence of battles or (c) presence of protests and riots. Points are regression coefficients, bars are 95% confidence intervals, and standard errors are clustered at the protected area level. The spatial unit of observation is the area within a protected area's boundaries plus a 25-kilometer buffer around a protected area's boundaries, and the temporal unit of observation is the year. In all regressions, the number of observations is 3,625 and the control variables are area fixed effects, year fixed effects, a third-order polynomial in precipitation in m, and 12 degree day bins (Materials and Methods). The dependent variable in each plot is an indicator that equals 1 if that specific type of conflict occurs within 25 km of a protected area that year and equals 0 otherwise. The ATTs corresponding to this figure are displayed in Table S3.

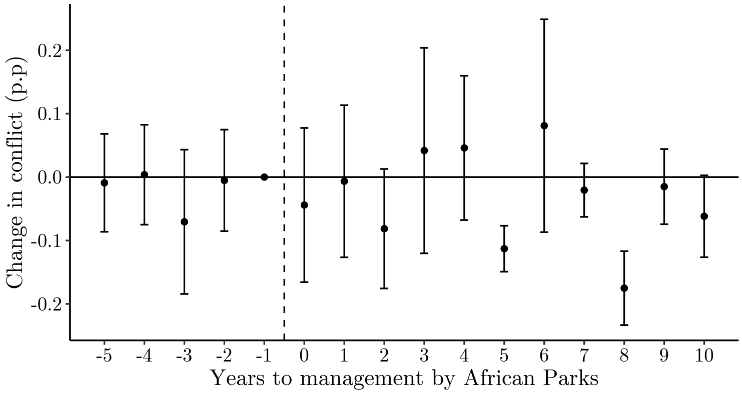

Fig. S14. AP management is unrelated to the presence of civilian targeting when Garamba National Park, Pendjari National Park, and W National Park are excluded from the sample. Points are regression coefficients, bars are 95% confidence intervals, and standard errors are clustered at the protected area level. The spatial unit of observation is the area within a protected area's boundaries plus a 25-kilometer buffer around a protected area's boundaries, and the temporal unit of observation is the year. The number of observations is 3,550 and the control variables are area fixed effects, year fixed effects, a third-order polynomial in precipitation in m, and 12 degree day bins (Materials and Methods). The dependent variable is an indicator that equals 1 if any civilian targeting occurred and equals 0 otherwise. The ATT is -0.015 and the ATT's standard error is 0.031. Garamba National Park, Pendjari National Park, and W National Park have been omitted from the sample to demonstrate how their inclusion likely drives the positive relationship between AP management and civilian targeting we observe in Figure S13a.

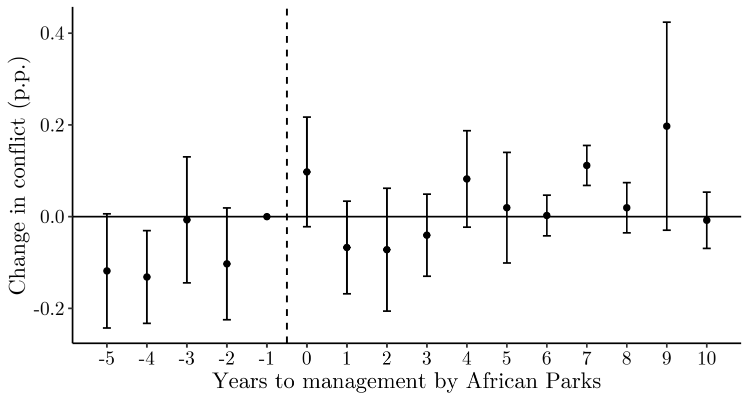

Fig. S15. AP management is unrelated to the presence of conflict events. Points are regression coefficients, bars are 95% confidence intervals, and standard errors are clustered at the protected area level. The spatial unit of observation is the area within a protected area's boundaries plus a 25-kilometer buffer around a protected area's boundaries, and the temporal unit of observation is the year. The number of observations is 3,625 and the control variables are area fixed effects, year fixed effects, a third-order polynomial in precipitation in m, and 12 degree day bins (Materials and Methods). The dependent variable is an indicator that equals 1 if any conflict events occurred and equals 0 otherwise. The mean of the dependent variable in the control group is 0.286. The ATT is 0.048 (standard error = 0.037).

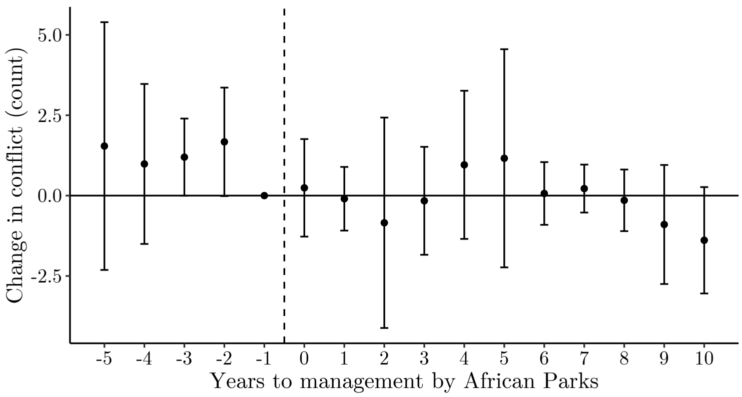

Fig. S16. AP management does not affect the number of civilian targeting events. Points are regression coefficients, bars are 95% confidence intervals, and standard errors are clustered at the protected area level. The spatial unit of observation is the area within a protected area's boundaries plus a 25-kilometer buffer around a protected area's boundaries, and the temporal unit of observation is the year. The number of observations is 3,625 and the control variables are area fixed effects, year fixed effects, a third-order polynomial in precipitation in m, and 12 degree day bins. The dependent variable is the number of civilian targeting events. The ATT is -0.186, the ATT's standard error is 0.535, and the mean of the dependent variable among the control group is 1.291.

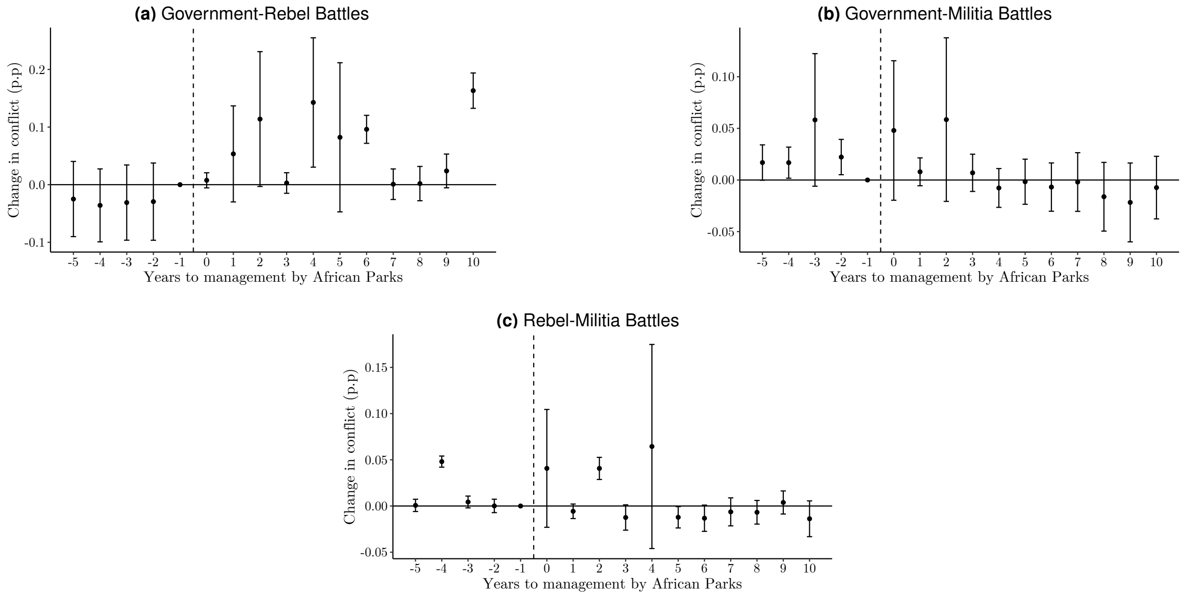

Fig. S17. AP management is associated with an increase in the presence of (a) battles between government and rebel forces but not (b) battles between government forces and local militias and (c) battles between rebel forces and local militias. Points are regression coefficients, bars are 95% confidence intervals, and standard errors are clustered at the protected area level. The spatial unit of observation is the area within a protected area's boundaries plus a 25-kilometer buffer around a protected area's boundaries, and the temporal unit of observation is the year. In all regressions, the number of observations is 3,625 and the control variables are area fixed effects, year fixed effects, a third-order polynomial in precipitation in m, and 12 degree day bins (Materials and Methods). The dependent variable in each plot is an indicator that equals 1 if that specific type of conflict occurs within 25 km of a protected area that year and equals 0 otherwise. The ATTs are (a) 0.065 (standard error = 0.022), (b) 0.017 (standard error = 0.011), and (c) 0.009 (standard error = 0.008). The means of the dependent variables among the control group are 0.043, 0.047, and 0.014, respectively.

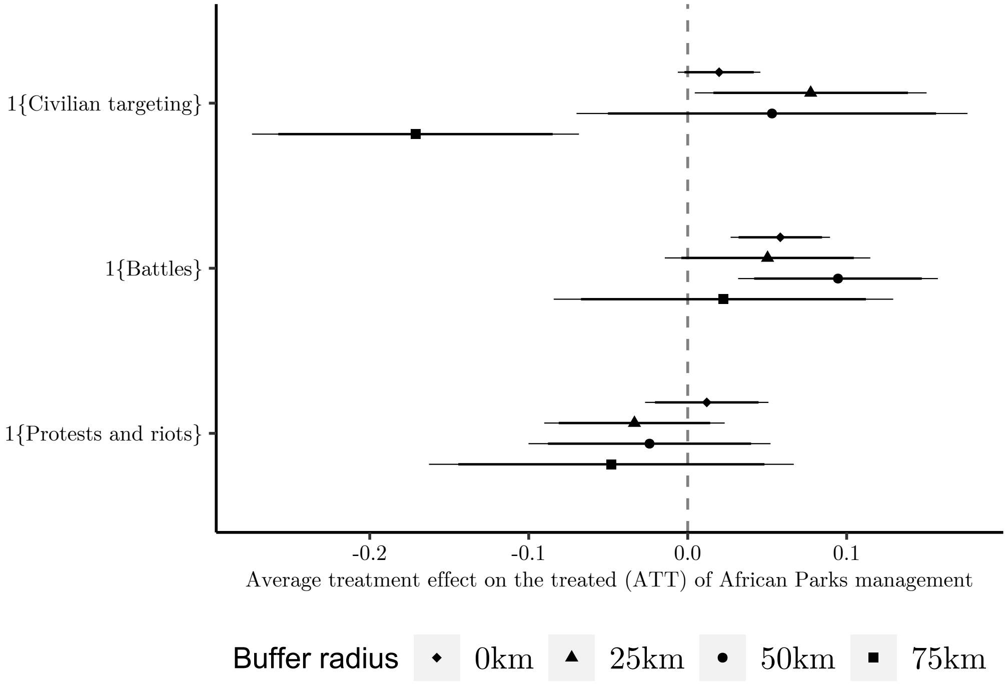

Fig. S18. Variation in the average treatment effect on the treated (ATT) of AP management on conflict across different buffer radii. Each point presents the result of a separate regression, where the number of observations is 3,625, the control variables are area fixed effects, year fixed effects, a third-order polynomial in precipitation in m, and 12 degree day bins, standard errors are clustered at the protected area level, and the outcome variable is listed on the y-axis. Points are ATT estimates, and thick and thin bars represent 90% and 95% confidence intervals, respectively. Here we vary the size of the buffer used to construct our spatial unit of observation from 25 kilometers to 0 kilometers, 50 kilometers, and 75 kilometers.

3. Supplementary Tables

	Area (km^2) (1)	Longitude (2)	Latitude (3)	DD $>$ 32 C (4)	Precip (m) (5)
Coefficient	1,220	0.855	3.968	30.227	-0.189
Standard Error	(2,820)	(2.399)	(2.831)	(15.176)	(0.159)
Observations	145	145	145	3,442	3,442
Year FE	No	No	No	Yes	Yes
Control Mean	7,669	24.298	-7.362	35.421	1.167

Table S1. Balance of treatment and control protected areas on observables. Each column displays the average difference between treatment (ever managed by AP) and control protected areas. The dependent variable being tested is specified in the column title. There is one observation per protected area in Columns 1 to 3. "DD $>$ 32 C" (Column 4) denotes degree days above 32 C and "Precip" denotes precipitation. Columns 4 and 5 use pre-period data for treatment areas and data from all years for control areas. Columns 4 and 5 control for year fixed effects and the standard errors are clustered at the level of protected area.

	Difference	Control Mean	Observations
**A. Outcomes**			
PIKE (elephant poaching)	0.179	0.437	523
	(0.078)		
log(bird abundance)	-0.215	3.003	139,925
	(0.104)		
1{iNaturalist tourism}	-0.131	0.462	3,442
	(0.057)		
1{eBird tourism}	-0.227	0.510	3,442
	(0.078)		
1{Civilian targeting}	0.025	0.163	3,442
	(0.077)		
1{Battles}	0.073	0.107	3,442
	(0.073)		
1{Protests and riots}	0.020	0.170	3,442
	(0.065)		
Standardized asset wealth	-0.224	0.034	2,594
	(0.113)		
**B. Mechanisms**			
Design and Planning (normalized)	-0.311	0.044	146
	(0.235)		
Capacity and Resources (normalized)	-0.133	-0.005	147
	(0.308)		
Monitoring and Enforcement Systems (normalized)	-0.079	0.013	147
	(0.262)		
Decision-Making Inclusiveness (normalized)	-0.367	0.037	145
	(0.242)		

Table S2. Tests for differences in pre-period outcomes and mechanisms. Each column displays the average difference between treatment (ever managed by AP) and control protected areas. The dependent variable being tested is specified in the column title. The three conflict variables and standardized asset wealth are calculated for a 25 km buffer around protected areas (inclusive of the protected area itself); all other variables only include data inside protected areas. All columns use pre-period data for treatment areas and data from all years for control areas. All columns control for year fixed effects and the standard errors are clustered at the level of protected area.

Dependent Variable (1)	ATT (2)	Standard Error (3)	N (4)	Control Mean (5)
**Wildlife Outcomes**				
PIKE (elephant poaching)	-0.153	(0.069)	578	0.437
log(bird abundance)	0.318	(0.072)	145,200	3.003
**Tourism**				
1{iNaturalist tourism}	0.215	(0.033)	3,625	0.462
1{eBird tourism}	0.190	(0.108)	3,625	0.510
**Economic Development**				
Standardized asset wealth	0.102	(0.034)	2,755	0.034
**Conflict**				
1{Civilian targeting}	0.077	(0.037)	3,625	0.163
1{Battles}	0.050	(0.033)	3,625	0.107
1{Protests and riots}	-0.033	(0.029)	3,625	0.170

Table S3. Average effect of AP management on wildlife, tourism, economic development, and conflict outcomes. Each row presents the result of a separate regression. Column 1 specifies the dependent variable in each regression. The Average Treatment effect on the Treated (ATT) in Column 2 is the average effect of AP management on a given dependent variable. Column 3 displays the ATT's standard error. Standard errors are clustered at the level of protected area. Column 4 reports the number of observations in the regression and Column 5 shows the mean of the dependent variable among control group protected areas.

Cohort	Coefficient	Standard error	Treated N	Control mean
**A. Average treatment effect on the treated by cohort**				
2003	-1.291	(0.083)	604	2.188
2008	-0.386	(0.053)	87	2.188
2010	0.092	(0.103)	1,850	2.188
2015	0.168	(0.055)	839	2.188
2017	0.384	(0.185)	123	2.188
2019	-0.133	(0.074)	54	2.188
2020	0.049	(0.051)	1,502	2.188
2021	0.193	(0.044)	1,234	2.188
**B. Average treatment effect on the treated (all cohorts)**				
All	-0.107	(0.053)	7,574	2.188

Table S4. Average effects of AP management on log(bird species). This table displays average treatment effects on the treated (ATTs) derived from the regression described in SI B.5. The dependent variable in this regression is the log(number of unique bird species observed) per birding trip. Panel A displays all ATTs returned from summarizing the regression object by the calendar year in which protected areas were transferred to AP ("cohort"). The command in R, summary(regression object, agg = "cohort"), returns ATTs for 8 of 12 possible cohorts, perhaps due to insufficient data or variation in the data for the remaining 4 cohorts. Panel B displays the ATT across all parks managed by AP. The Coefficient displays a given ATT and the Standard error column displays the ATT's standard error. The Treated N column is the number of observations in the given AP cohort (Panel A) or across all parks managed by AP (Panel B). The total number of observations in the regression is 145,200. The Control mean column is the mean log(number of unique bird species observed) across all control parks.

Table S5: List of protected areas included in analysis.

Name	Country	Group
National Park Iona	Angola	Treatment
National Park Cameia	Angola	Control
Integral Nature Reserve and the Luando	Angola	Control
Luengue-Luiana National Park	Angola	Control
Mavinga National Park	Angola	Control
W (Benin)	Benin	Treatment
Boucle de la Pendjari	Benin	Treatment
Chobe	Botswana	Control
Makgadikgadi Pans	Botswana	Control
Gemsbok	Botswana	Control
Central Kalahari	Botswana	Control
W du Burkina Faso	Burkina Faso	Control
Arly	Burkina Faso	Control
Bouba Ndjida	Cameroon	Control
Dja	Cameroon	Control
Faro	Cameroon	Control
Campo-Ma'an	Cameroon	Control
Lobéké	Cameroon	Control
Mbam et Djerem	Cameroon	Control
Boumba Bek	Cameroon	Control
Chinko	Central African Republic	Treatment
Bamingui-Bangoran	Central African Republic	Control
Vassako-Bolo	Central African Republic	Control
Andre Felix	Central African Republic	Control
Manovo-Gounda St Floris National Park	Central African Republic	Control
Dzanga-Ndoki	Central African Republic	Control
Zakouma	Chad	Treatment
Ennedi Natural and Cultural Reserve	Chad	Treatment
Siniaka-Minia	Chad	Treatment
Taï National Park	Côte d'Ivoire	Control
Comoe National Park	Côte d'Ivoire	Control
Garamba National Park	Democratic Republic of Congo	Treatment
Maiko	Democratic Republic of Congo	Control
Kundelungu	Democratic Republic of Congo	Control
Bili-Uere	Democratic Republic of Congo	Control
Virunga	Democratic Republic of Congo	Control
Lomami National Park	Democratic Republic of Congo	Control
Salonga	Democratic Republic of Congo	Control
Dahlak Island PA	Eritrea	Control
Bale Mountains	Ethiopia	Control
Gambella	Ethiopia	Control
Chebera Churchura	Ethiopia	Control
Alitash	Ethiopia	Control
Wongha-Wonghé	Gabon	Control
Minkebe	Gabon	Control
Ivindo	Gabon	Control
Loango	Gabon	Control
Lopé	Gabon	Control
Plateaux Batéké	Gabon	Control
Mole	Ghana	Control
Bolama - Bijagos	Guinea-Bissau	Control
Tsavo East	Kenya	Control
Marsabit	Kenya	Control
Aberdare	Kenya	Control
Buffalo Springs	Kenya	Control
Samburu	Kenya	Control
Shaba	Kenya	Control
Tsavo West	Kenya	Control

Mount Kenya National Park/Natural Forest	Kenya	Control
Sapo National Park	Liberia	Control
Masoala	Madagascar	Control
Liwonde National Park	Malawi	Treatment
Nkhotakota Wildlife Reserve	Malawi	Treatment
Majete Wildlife Reserve	Malawi	Treatment
Mangochi	Malawi	Treatment
Nyika National Park	Malawi	Control
Kasungu National Park	Malawi	Control
Vwaza Marsh Wildlife Reserve	Malawi	Control
Bazaruto	Mozambique	Treatment
Banhine	Mozambique	Control
Zinave	Mozambique	Control
Gorongosa	Mozambique	Control
Maputo	Mozambique	Control
Quirimbas	Mozambique	Control
Limpopo	Mozambique	Control
Gilé	Mozambique	Control
Niassa	Mozambique	Control
Namib-Naukluft	Namibia	Control
Skeleton Coast Park	Namibia	Control
Ai-Ais Hot Springs	Namibia	Control
Khaudum	Namibia	Control
Etosha Pan, Lake Oponono & Cuvelai drainage	Namibia	Control
Bwabwata	Namibia	Control
Kainji Lake	Nigeria	Control
Gashaka-Gumti	Nigeria	Control
Cross River	Nigeria	Control
Yankari	Nigeria	Control
Odzala Kokoua	Republic of Congo	Treatment
Lac Télé	Republic of Congo	Control
Akagera	Rwanda	Treatment
Nyungwe	Rwanda	Treatment
Niokolo-Koba National Park	Senegal	Control
Kruger National Park	South Africa	Control
Kalahari Gemsbok National Park	South Africa	Control
Addo-Elephant National Park	South Africa	Control
Karoo National Park	South Africa	Control
Augrabies Falls National Park	South Africa	Control
Pilanesberg National Park	South Africa	Control
Richtersveld National Park	South Africa	Control
Tankwa-Karoo National Park	South Africa	Control
Madikwe Nature Reserve	South Africa	Control
Marakele National Park	South Africa	Control
uKhahlamba-Drakensberg Park	South Africa	Control
iSimangaliso Wetland Park	South Africa	Control
Table Mountain National Park	South Africa	Control
Namaqua National Park	South Africa	Control
Sederberg Wilderness Area	South Africa	Control
Boma	South Sudan	Treatment
Badingilo	South Sudan	Treatment
Southern	South Sudan	Control
Sudd	South Sudan	Control
Dinder	Sudan	Control
Serengeti National Park	Tanzania	Control
Ruaha National Park	Tanzania	Control
Tarangire National Park	Tanzania	Control
Katavi National Park	Tanzania	Control
Kilimanjaro National Park	Tanzania	Control
Mkomazi National Park	Tanzania	Control
Ngorongoro Conservation Area	Tanzania	Control

Selous Game Reserve	Tanzania	Control
Saadani National Park	Tanzania	Control
Moyowosi G.R (N)	Tanzania	Control
Mahale Mts.National Park	Tanzania	Control
Udzungwa Mountain	Tanzania	Control
Kitulo Plateau National Park	Tanzania	Control
Mount Rungwe	Tanzania	Control
Fazao-Malfakassa	Togo	Control
Murchison Falls	Uganda	Control
Queen Elizabeth	Uganda	Control
Kidepo Valley	Uganda	Control
Kafue	Zambia	Treatment
Liuwa Plain	Zambia	Treatment
Bangweulu	Zambia	Treatment
South Luangwa	Zambia	Control
North Luangwa	Zambia	Control
Lukusuzi	Zambia	Control
Nsumbu	Zambia	Control
Lower Zambezi	Zambia	Control
Matusadona	Zimbabwe	Treatment
Chizarira	Zimbabwe	Control
Matopos	Zimbabwe	Control
Hwange	Zimbabwe	Control
Zambezi	Zimbabwe	Control
Mana Pools	Zimbabwe	Control
Chimanimani	Zimbabwe	Control

Cohort	Coefficient	Standard error	Treated N	Control mean
**A. Average treatment effect on the treated by cohort**				
2005	-0.068	(0.091)	19	0.437
2010	-0.181	(0.113)	42	0.437
2017	-0.213	(0.094)	17	0.437
2020	-0.309	(0.109)	14	0.437
2021	-0.207	(0.087)	4	0.437
**B. Average treatment effect on the treated (all cohorts)**				
All	-0.153	(0.069)	99	0.437

Table S6. Average effects of AP management on elephant poaching. This table displays average treatment effects on the treated (ATTs) derived from the regression described in SI A.1. The dependent variable in this regression is the Proportion of Illegally Killed Elephants (PIKE). Panel A displays all ATTs returned from summarizing the regression object by the calendar year in which protected areas were transferred to AP ("cohort"). The command in R, summary(regression object, agg = "cohort"), returns ATTs for 5 of 6 possible cohorts, perhaps due to insufficient data or variation in the data for the remaining cohort. For comparison, Panel B displays the ATT across all protected areas managed by AP. The Coefficient displays a given ATT and the Standard error column displays the ATT's standard error. The Treated N column is the number of observations in the given AP cohort (Panel A) or across all protected areas managed by AP (Panel B). The total number of observations in the regression is 578. The Control mean column is the mean PIKE across all control protected areas.

Cohort	Coefficient	Standard error	Treated N	Control mean
**A. Average treatment effect on the treated by cohort**				
2003	-0.369	(0.126)	604	3.003
2008	-1.100	(0.072)	87	3.003
2010	0.474	(0.139)	1,850	3.003
2015	0.546	(0.071)	839	3.003
2017	-0.691	(0.581)	123	3.003
2019	-0.377	(0.081)	54	3.003
2020	0.170	(0.079)	1,502	3.003
2021	0.785	(0.057)	1,234	3.003
**B. Average treatment effect on the treated (all cohorts)**				
All	0.318	(0.072)	7,574	3.003

Table S7. Average effects of AP management on bird abundances. This table displays average treatment effects on the treated (ATTs) derived from the regression described in SI A.2. The dependent variable in this regression is the log(bird count) per birding trip. Panel A displays all ATTs returned from summarizing the regression object by the calendar year in which protected areas were transferred to AP ("cohort"). The command in R, summary(regression object, agg = "cohort"), returns ATTs for 8 of 12 possible cohorts, perhaps due to insufficient data or variation in the data for the remaining 4 cohorts. For comparison, Panel B displays the ATT across all protected areas managed by AP. The Coefficient displays a given ATT and the Standard error column displays the ATT's standard error. The Treated N column is the number of observations in the given AP cohort (Panel A) or across all protected areas managed by AP (Panel B). The total number of observations in the regression is 145,200. The Control mean column is the mean log(bird count) across all control protected areas.

References

1. Monitoring the Illegal Killing of Elephants (2023) Numbers of carcasses found for each site and year. Downloaded on Jan 16, 2023. Available at https://cites.org/sites/default/files/MIKE/data/Numbers_Carcasses_eachMIKESite_year_2022-03-31.xlsx.
2. Burn RW, Underwood FM, Blanc J (2011) Global trends and factors associated with the illegal killing of elephants: a hierarchical Bayesian analysis of carcass encounter data. *PLoS one* 6(9):e24165.
3. Hsiang S, Sekar N (2016) Does Legalization Reduce Black Market Activity? Evidence from a Global Ivory Experiment and Elephant Poaching Data (National Bureau of Economic Research Working Paper 22314).
4. Kahindi O, et al. (2010) Employing participatory surveys to monitor the illegal killing of elephants across diverse land uses in Laikipia–Samburu, Kenya. *African Journal of Ecology* 48(4):972–983.
5. Jachmann H (2012) Pilot study to validate PIKE-based inferences at site level. *Pachyderm* 52:72–87.
6. Rambachan A, Roth J (2023) A more credible approach to parallel trends. *Review of Economic Studies* 90(5):2555–2591.
7. Cornell Lab of Ornithology (2023) eBird Basic Dataset. Version: EBD_relFeb-2023. Downloaded on April 10, 2023.
8. Liang Y, et al. (2020) Conservation cobenefits from air pollution regulation: Evidence from birds. *Proceedings of the National Academy of Sciences* 117(49):30900–30906.
9. Sun L, Abraham S (2021) Estimating dynamic treatment effects in event studies with heterogeneous treatment effects. *Journal of Econometrics* 225(2):175–199.
10. iNaturalist (2023) Dataset. Downloaded on March 1, 2023. Available from https://www.inaturalist.org.
11. Jean N, et al. (2016) Combining satellite imagery and machine learning to predict poverty. *Science* 353(6301):790–794.
12. Yeh C, et al. (2020) Using publicly available satellite imagery and deep learning to understand economic well-being in Africa. *Nature Communications* 11(1):2583.
13. Raleigh C, Kishi R, Linke A (2023) Political instability patterns are obscured by conflict dataset scope conditions, sources, and coding choices. *Humanities and Social Sciences Communications* 10(1):1–17.
14. Stanton JA (2015) Regulating militias: Governments, militias, and civilian targeting in civil war. *Journal of Conflict Resolution* 59(5):899–923.
15. Asal V, Phillips BJ, Rethemeyer RK, Simonelli C, Young JK (2019) Carrots, sticks, and insurgent targeting of civilians. *Journal of Conflict Resolution* 63(7):1710–1735.
16. Schouten P, Murairi J, Kubuya S (2017) "Everything that moves will be taxed": The Political Economy of Roadblocks in North and South Kivu.
17. Matthysen K, Gobbers E (2022) Armed conflict, insecurity, and mining in Eastern DRC: Reflections on the nexus between natural resources and armed conflict.
18. Nellemann C, Stock J, Shaw M (2018) *World Atlas of Illicit Flows.* (Global Initiative).
19. United Nations Counter-Terrorism Committee Executive Directorate (2022) Concerns over the use of proceeds from the exploitation, trade, and trafficking of natural resources for the purposes of terrorism financing.
20. Höije K (2014) Rebels Retain Control of Rich Mine in Central African Republic.
21. Ntanyoma DR (2022) M23: Four things you should know about the rebel group's campaign in Rwanda-DRC conflict.
22. McGuirk EF, Nunn N (2021) Transhumant Pastoralism, Climate Change, and Conflict in Africa (National Bureau of Economic Research Working Paper 28243).
23. Parker DP, Vadheim B (2017) Resource cursed or policy cursed? US regulation of conflict minerals and violence in the Congo. *Journal of the Association of Environmental and Resource Economists* 4(1):1–49.

24. O'loughlin J, et al. (2012) Climate variability and conflict risk in East Africa, 1990–2009. *Proceedings of the National Academy of Sciences* 109(45):18344–18349.
25. Schon J, Koren O (2022) Introducing AfroGrid, a unified framework for environmental conflict research in Africa. *Scientific Data* 9(1):116.
26. Stearns J, Vogel C (2017) The Landscape of Armed Groups in Eastern Congo: Fragmented, politicized networks.
27. OECD and Sahel and West Africa Club (2023) *Urbanisation and Conflicts in North and West Africa.*
28. Coad L, et al. (2015) Measuring impact of protected area management interventions: current and future use of the Global Database of Protected Area Management Effectiveness. *Philosophical Transactions of the Royal Society B: Biological Sciences* 370(1681):20140281.
29. Stolton S, et al. (2007) *Reporting progress in protected areas: A site-level management effectiveness tracking tool (2nd ed.).* (World Bank/WWF Forest Alliance, Gland, Switzerland).
30. Geldmann J, et al. (2018) A global analysis of management capacity and ecological outcomes in terrestrial protected areas. *Conservation Letters* 11(3):e12434.
31. Geldmann J, Manica A, Burgess ND, Coad L, Balmford A (2019) A global-level assessment of the effectiveness of protected areas at resisting anthropogenic pressures. *Proceedings of the National Academy of Sciences* 116(46):23209–23215.
